# Improving obesity management: Insights from the ACTION Switzerland survey of people with obesity, physicians and dietitians

**DOI:** 10.1111/cob.12716

**Published:** 2024-11-07

**Authors:** Dominique Durrer, Patrick Pasi, Ralph Peterli, Doris Fischer‐Taeschler, Gabriela Fontana, Gionata Cavadini, Philipp A. Gerber

**Affiliations:** ^1^ Obesity Centre, Eurobesitas EASO COMs Centre Vevey Switzerland; ^2^ Department of Consultation‐Liaison Psychiatry and Psychosomatic Medicine, University Hospital Zürich University of Zürich Zürich Switzerland; ^3^ Department of Visceral Surgery, Clarunis University Digestive Health Care Center, St. Clara Hospital and University Hospital Basel Basel Switzerland; ^4^ Swiss Obesity Alliance Baden Switzerland; ^5^ Clinical, Medical and Regulatory, Novo Nordisk Pharma AG Zürich Switzerland; ^6^ Department of Endocrinology, Diabetology and Clinical Nutrition University Hospital Zürich (USZ) and University of Zürich (UZH) Zürich Switzerland

**Keywords:** behaviour, clinical care, obesity management, patient education, physician attitudes, weight bias

## Abstract

ACTION Switzerland (NCT05232786) examined obesity‐related perceptions, attitudes, behaviours and potential barriers to treatment among people with obesity (PwO) and healthcare professionals (HCPs). In March/April 2022, adult PwO (body mass index ≥30 kg/m^2^, per self‐reported height/weight) and physicians/certified dietitians who manage PwO in Switzerland completed online surveys in a cross‐sectional design. Overall, 1002 PwO, 125 physicians and 25 dietitians completed the survey. Most physicians (97%) and dietitians (100%), but only 57% of PwO, recognized obesity as a chronic disease. Only 42% of PwO considered themselves to have obesity/extreme obesity, while 61% who had discussed weight with an HCP reported receiving an obesity diagnosis. Many PwO (76%) believed weight loss was entirely their responsibility; physicians were less likely than dietitians to agree it was completely their patients' responsibility (28% vs. 68%). Physicians and dietitians report primarily initiating conversations about weight when patients have obesity‐related comorbidities (85% and 64%); their top reasons for not discussing obesity were patients' perceived lack of motivation (76% and 60%) or interest (72% and 64%) in losing weight. In conclusion, some PwO are not aware that obesity is a chronic disease and incorrectly assume complete responsibility for weight loss. Improved communication between PwO and HCPs is required.

## Graphical abstract

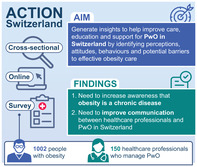

**What is already known about this subject**

- Many people with obesity (PwO) experience weight bias and stigma, including within the healthcare setting, which may act as a barrier to effective obesity management.
- Perceptions, attitudes, behaviours, and potential barriers to effective obesity care have been explored in previous survey studies, including the ACTION International Observation study of PwO and healthcare professionals (HCPs), but there have been no such studies in Switzerland. Additionally, no previous ACTION studies have surveyed dietitians.

**What this study adds**

- Our results suggest that in Switzerland, perceptions, attitudes, behaviours and barriers to effective obesity care are similar to those in the international community. There is a need to increase awareness that obesity is a chronic disease and improve communication and collaboration during treatment.
- Additionally, we found that dietitians are eager to be involved early in the treatment process. They schedule more follow‐up appointments than physicians and are more likely to believe behaviour therapy is effective for weight management.
- Enhanced collaboration between physicians and dietitians at an earlier stage following obesity diagnosis and use of behavioural interventions may decrease stigma in healthcare settings and help HCPs support patients to achieve their long‐term weight‐management goals.

## 1 INTRODUCTION

Obesity increases the risk of many adverse health conditions and is associated with an increased risk of mortality.^1^, ^2^ Despite growing recognition that obesity is a chronic disease requiring long‐term management,^3^ the prevailing cultural narrative frames obesity as a matter of personal responsibility and overlooks the role of biological and environmental factors that are not fully within an individual's control.^4^ As a result, many people with obesity (PwO) experience weight bias and stigma,^5^, ^6^ including within the healthcare setting, which may act as a barrier to the provision of effective medical care and obesity management.^7^

To improve care, education and support for PwO, it is important to understand the perceptions, attitudes and behaviours of PwO and the healthcare professionals (HCPs) who treat them. These have been explored in previous survey‐based studies, including the Awareness, Care, and Treatment In Obesity maNagement (ACTION) studies conducted in the United States, Canada and Denmark, and the ACTION International Observation and Asia‐Pacific studies conducted in 11 and 9 countries, respectively.^8^, ^9^, ^10^, ^11^, ^12^ However, there have been no similar survey‐based studies in Switzerland, where 12% of the population had obesity in 2022^13^ and an estimated 37% of adults will have obesity by 2035.^14^ Furthermore, the HCPs surveyed in previous ACTION studies did not include certified dietitians – a group that plays an equally important role in obesity management.

The ACTION Switzerland study aimed to identify perceptions, attitudes, behaviours and potential barriers to effective obesity care among PwO and HCPs, including physicians and dietitians, in Switzerland. The insights generated may help to guide collaborative action to improve care, education and support for PwO and HCPs in Switzerland.

## 2 MATERIALS AND METHODS

### 2.1 Study design and participants

ACTION Switzerland was a cross‐sectional, survey‐based study. The study is registered with ClinicalTrials.gov (NCT05232786).

A third‐party vendor (KJT Group Inc., Rochester, NY, USA) recruited prospective participants from online panels in Switzerland and collected online survey data between 8 March and 8 April 2022. A stratified general population sample from online consumer panels was screened to identify adult PwO, while HCPs were recruited from online HCP panels. Custom telephone recruitment (conducted by the online panel company) was also used for dietitians.

PwO were eligible if they lived in Switzerland, were aged ≥18 years and had a current body mass index (BMI) ≥30 kg/m^2^ (based on self‐reported height and weight). Exclusion criteria included current pregnancy; significant, unintentional weight loss (e.g. due to major injury/illness) in the past 6 months; and participation in intense fitness/bodybuilding programmes.

Eligible HCPs were physicians or certified dietitians who had been in clinical practice for 2 or more years, practised in Switzerland and spent at least 50% of their time directly caring for patients. Physicians were primary care practitioners whose speciality was family practice, general practice or general internal medicine (with a focus on primary care), or non‐primary care practitioners with relevant specialities, e.g. obstetrics/gynaecology, endocrinology/diabetology, psychiatry, general internal medicine (with a focus on diabetes and/or obesity) or bariatric surgery (although bariatric surgeons must have been involved in the management of obesity in addition to surgery). All physicians except psychiatrists were required to have seen ≥100 patients in the past month. Additionally, all HCPs were required to have seen patients with obesity, i.e. BMI ≥30 kg/m^2^, in the past month (≥5 patients for dietitians and psychiatrists; ≥10 patients for other HCPs).

Electronic informed consent was obtained from all respondents. An independent ethics committee (Kantonale Ethikkommission Zürich, Stampfenbachstrasse 121, 8090 Zürich, Switzerland) advised that ethical approval was not required for this study as it did not fall within the scope of the Human Research Act (reference number: BASEC‐Nr. Req‐2022‐00003).

This study was conducted in accordance with the Declaration of Helsinki, Swiss Data Protection Regulations, European Union General Data Protection Regulation and EphMRA Code of Conduct.

### 2.2 Survey development

Two online surveys were specifically developed for this study: one for PwO and one for HCPs (see Data S1 for survey questions). Wherever possible, survey themes were mirrored across respondent groups. Surveys were based on the ACTION International Observation surveys,^10^ but were modified with insights from an external steering committee (comprising HCPs and subject matter experts); notably, additional questions about weight stigma were added.

### 2.3 Procedures

KJT Group was responsible for data collection, management and reporting. Surveys were programmed using Decipher Survey Software (Forsta, Stamford, CT, USA) and hosted on a secure website.

Prospective participants were sent a unique survey link via email and completed an eligibility screener (see Data S1 for survey invites). To reduce bias, email invitations for PwO did not specify the topic of the survey and screening questions were designed to conceal the purpose of the study.

Eligible participants could continue to the online survey, which was provided in German, French, Italian and English. It was not possible to skip survey questions, so there were no missing data in completed surveys.

### 2.4 Outcomes

Primary outcomes included weight‐loss motivators, barriers and management; the proportion of PwO who made a serious weight‐loss effort; attitudes towards obesity and weight management; and interactions between PwO and HCPs. Surveys utilized single‐ or multiple‐item selection from a defined list, yes/no responses, 5‐point Likert scales and numeric entry fields.

### 2.5 Sample size

Sample sizes were chosen to reflect the population size of PwO in Switzerland, while balancing recruitment feasibility with statistical power. The target was 1150 completed surveys, comprising responses from 1000 PwO and 150 HCPs (75 primary care practitioners, 50 non‐primary care practitioner specialists and 25 dietitians).

### 2.6 Analysis

Analyses were based on data from respondents who completed the survey. There were three analysis sets: PwO, physicians and dietitians.

KJT Group analysed de‐identified data using SPSS (version 23.0; IBM, Armonk, NY, USA), Stata® (version IC 14.2; StataCorp LLC, College Station, TX, USA), Excel (Microsoft, Redmond, WA, USA) and Q Research Software (Displayr Inc., NSW, Australia). Results were reported as means, medians and proportions; for continuous data, outliers were truncated to the median, where appropriate. Proportions did not always sum to 100% due to rounding. For 5‐point Likert scales, some response options (e.g. the top or bottom two) were merged.

Weights were applied to data collected from PwO to mitigate selection bias and increase the generalizability of results. The final sample, including those who were not eligible for the study, was weighted on key demographics (sex, age, language region, education and household income) using general population targets from government and other public data. Data collected from HCPs were not weighted. The Data S1 contains additional information about the methods.

## 3 RESULTS

### 3.1 Participant characteristics

Overall, 1002 PwO, 125 physicians and 25 dietitians completed the survey (shown in Figure S1), which was in line with the target sample size; response rates were 4.2% (estimated), 13.7% and 10.2%, respectively. Median response times were 23, 33 and 32 min, respectively.

Participant demographics and characteristics are reported in Table 1. A subset of PwO reported being diagnosed with hypertension (35%), depression/anxiety (21%) or high cholesterol (17%), among other medical conditions; 29% reported having no comorbidities (Table 1). More details on reported comorbidities are summarized in Table S1. Physician specialities are reported in Table S2. Among the dietitians surveyed, 28% reported being self‐employed; the remainder worked in a public/private hospital (72%).

**TABLE 1:** Demographics and characteristics.

	PwO	Physicians	Dietitians
Full sample, *N*	1002	125	25
Age in years, mean (SD)	44.9 (15.1)	50.2 (9.2)	40.4 (10.9)
Sex,^a^ *n* (%)			
Male	495 (49)	75 (60)	2 (8)
Female	506 (50)	49 (39)	23 (92)
Other	1 (<1)	1 (1)	0
Region by language spoken, *n* (%)			
German	633 (63)	102 (82)	25 (100)
French	300 (30)	23 (18)	0
Italian	69 (7)	0	0
BMI of PwO,^b^ kg/m^2^			
<25 (not overweight)	N/A	32% (SD: 16)	24% (SD: 16)
25–29.9 (overweight)	N/A	31% (SD: 12)	19% (SD: 13)
30–34.9 (obesity class I)	59% (*n* = 596)	19% (SD: 8)	28% (SD: 15)
35–39.9 (obesity class II)	23% (*n* = 226)	12% (SD: 8)	21% (SD: 11)
≥40 (obesity class III)	18% (*n* = 180)	6% (SD: 6)	8% (SD: 8)
Number of comorbidities reported, %			
0	29	N/A	N/A
1	24	N/A	N/A
2	20	N/A	N/A
3	14	N/A	N/A
≥4	13	N/A	N/A
HCP experience, mean (SD)			
Years in practice	N/A	15.6 (8.2)	15.6 (12.2)
Years providing obesity care to patients	N/A	9.7 (7.1)	11.9 (8.7)
PwO seen in past month	N/A	62 (44.1)	50 (26.6)
Percentage of adult patients primarily seen for obesity	N/A	20 (19.0)	53 (22.0)

Abbreviations: BMI, body mass index; HCP, healthcare professional; N/A, not applicable; PwO, people with obesity; SD, standard deviation.

^a^ Based on responses to PwO S4 (‘Which gender do you identify with?’) and HCP S2 (‘Are you…?’); survey questions and response options are available in the Data S1.

^b^ BMI of recruited PwO and the PwO treated by recruited HCPs. PwO data are the percentage (*n*) of PwO in each BMI range; physician and dietitian data are the mean percentage (SD) of their PwO patients in each BMI range.

### 3.2 Attitudes and perceptions

**FIGURE 1:**
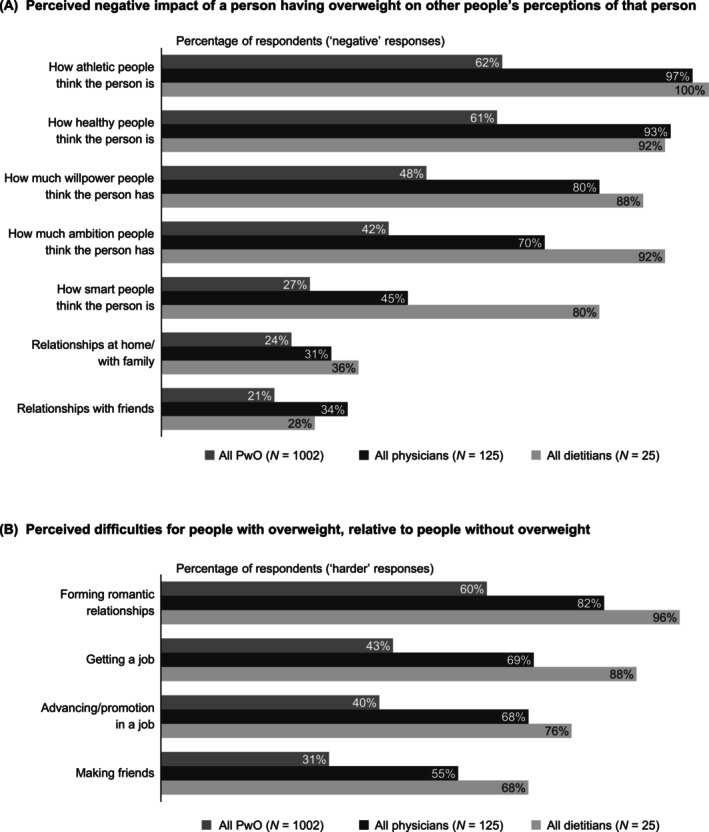
Weight stigma. In panel (A), data are the proportion of PwO, physicians and dietitians who indicated that a person having overweight had a ‘somewhat’ or ‘very’ negative impact on each prespecified response option, i.e. selected one of the bottom two response options from a 5‐point Likert scale where 1 meant ‘very negative impact’ and 5 meant ‘very positive impact’ (Data S1 survey questions: PwO Q425; HCP Q415). In panel (B), data are the proportion of PwO, physicians and dietitians who indicated that each prespecified response option was ‘somewhat’ or ‘much’ harder for a person with overweight, relative to a person without overweight, i.e. selected one of the bottom two response options from a 5‐point Likert scale where 1 meant ‘much harder’ and 5 meant ‘much easier’ (PwO Q420; HCP Q410). HCP, healthcare professional; PwO, people with obesity.

Many respondents thought that excess weight has a somewhat/very negative impact on other people's perceptions of a person and that it would be somewhat/much harder for a person with overweight to form romantic relationships, get a job, be promoted and make friends than a person without overweight (shown in Figure 1). Additionally, most PwO, physicians and dietitians felt that obesity has an extreme impact on a person's overall health (81%, 66% and 60%, respectively).

**FIGURE 2:**
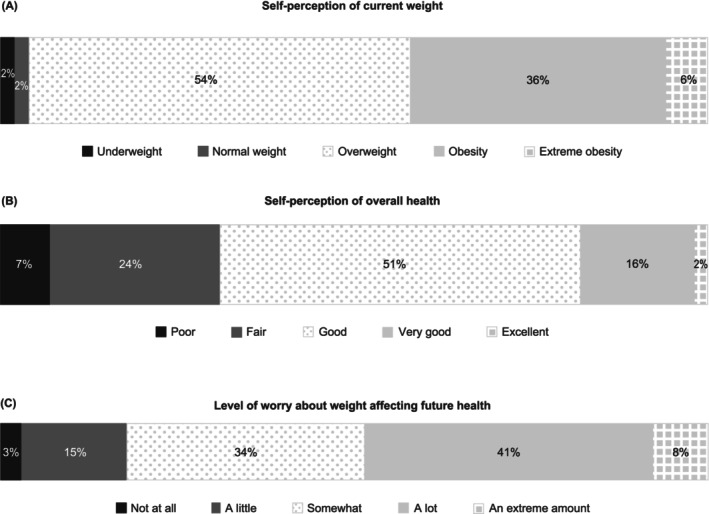
PwO self‐perceptions and level of worry about their weight. Proportion of PwO (*N* = 1002) who selected each prespecified response option (Data S1 survey questions: PwO Q103, Q102A and Q415). Percentages may not sum to 100% due to rounding. PwO, people with obesity.

Most PwO underestimated their own weight classification. Additionally, most PwO believed that their health was good, very good or excellent; despite this, most were at least somewhat worried that their weight may affect their future health (shown in Figure 2).

**FIGURE 3:**
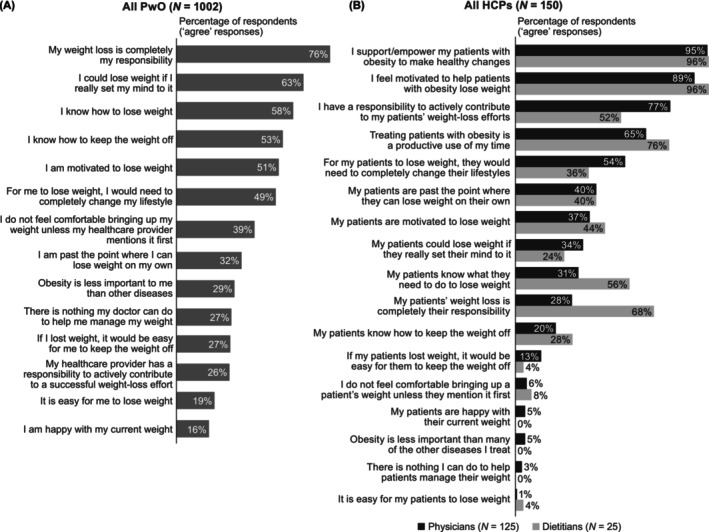
Attitudes towards weight loss. Proportion of PwO, physicians and dietitians who agreed with each statement, i.e. selected one of the top two response options from a 5‐point Likert scale where 1 meant ‘do not agree at all’ and 5 meant ‘completely agree’ (Data S1 survey questions: PwO Q500; HCP Q503). HCP, healthcare professional; PwO, people with obesity.

Only 57% of PwO, but nearly all HCPs (physicians: 97%; dietitians: 100%), recognized that obesity is a chronic disease (shown in Figure S2). Additionally, most respondents agreed that obesity treatment should be a team effort between different HCPs. Despite this, 76% of PwO agreed that weight loss was entirely their own responsibility (shown in Figure 3). By comparison, only 28% of physicians agreed that weight loss was entirely their patients' responsibility, and 77% agreed that they have a responsibility to actively contribute to their patients' weight‐loss efforts. However, 68% of dietitians believed weight loss was entirely their patients' responsibility, and 52% agreed that they have a responsibility to actively contribute to their patients' weight‐loss efforts. Approximately half of PwO agreed that they are motivated to lose weight, but fewer HCPs agreed that their patients with obesity are motivated to lose weight (shown in Figure 3).

### 3.3 Conversations about weight

The mean age at which PwO began struggling with their weight was 31 years; on average, 5 years passed before an HCP first discussed their weight. Approximately half of PwO had discussed weight with an HCP in the past 5 years (54%) – most commonly their primary care physician, a dietitian or an obesity specialist (reported by 72%, 45% and 39%, respectively). By comparison, physicians and dietitians indicated that they discuss weight with 73% and 83% of their patients with obesity, on average. However, only 28% of PwO had spoken to their HCP about a weight‐loss plan within the past 6 months. Furthermore, only 61% of PwO who had discussed weight with an HCP reported that they had been diagnosed with obesity. By comparison, physicians and dietitians reported informing 79% and 73% of their patients with obesity about the diagnosis, on average.

The top criterion that physicians consider when initiating discussions about obesity with patients is the presence of obesity‐related comorbidities (selected by 85%). For dietitians, the top criteria were the presence of obesity‐related comorbidities and how receptive they expect the patient to be to the discussion (both selected by 64%). Physicians' top reasons for not initiating weight‐management discussions were the patient not feeling motivated (76%) or interested in losing weight (72%), and the appointment not being long enough (50%). Dietitians' top reasons were the patient not being interested in losing weight (64%) or feeling motivated (60%), and the patient already knowing what they need to do to manage their weight (52%). The top reasons for PwO not discussing weight with an HCP were believing it is their own responsibility to manage their weight (reported by 44%) and already knowing what they need to do to manage their weight (41%).

Among PwO whose HCP usually initiates conversations on the topic of weight (*n* = 259), 54% did not like that their HCP brings up their weight. By comparison, just over half of PwO whose HCP did not usually start the conversation or who had not discussed weight with an HCP in the past 5 years (*n* = 743) would like their HCP to bring up weight (53%). PwO who had discussed weight with an HCP felt a mixture of emotions after their most recent discussion – 67% reported at least one positive emotion, while 52% reported at least one negative emotion (shown in Figure S3). While 54% of PwO who had discussed weight with an HCP agreed that they felt comfortable talking to their HCP about their weight, only 45% of PwO who had not discussed weight would feel comfortable doing so.

### 3.4 Weight‐loss attempts/goals

Most PwO (*n =* 894; 89%) had made at least one serious weight‐loss attempt. However, many PwO struggled to lose weight and maintain weight loss; only 40% reported losing ≥5% of their body weight during the previous 3 years, of whom only 38% maintained the weight loss for at least 1 year (15% of all PwO; shown in Figure S4).

For PwO, the top motivators to lose weight were: wanting to feel better physically, have more energy or be more active; wanting to be more fit/in better shape; and having general health concerns (shown in Figure S5). PwO also identified several barriers to weight loss, most commonly lack of exercise, unhealthy eating habits and lack of motivation (shown in Figure S6).

PwO reported setting similar personal weight‐loss targets if they were making a weight‐loss attempt (equivalent to a 21.7% reduction in body weight, on average) to targets recommended by HCPs (21.5% reduction, on average, according to PwO who had discussed weight with an HCP and for whom weight loss had been recommended).

### 3.5 Weight‐management methods

PwO felt the best types of weight‐loss support were physical activity programmes, specific meal plans to follow and motivational programmes (selected by 31%, 29% and 27%, respectively). By comparison, physicians' top three were prescription weight‐loss drugs, physical activity programmes and bariatric surgery (selected by 58%, 53% and 52%, respectively), and dietitians' top three were meetings with a dietitian, physical activity programmes and motivational programmes (selected by 72%, 68% and 40%, respectively).

**FIGURE 4:**
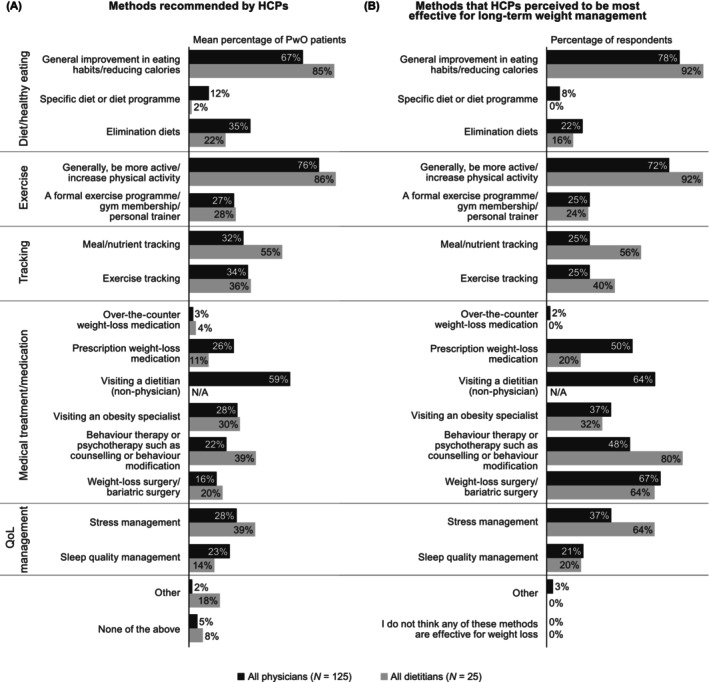
Weight‐management methods: HCP perspective. In panel (A), data are the mean percentage of patients with obesity to whom physicians and dietitians recommend each of the prespecified options in the survey question HCP Q128 (see Data S1). In panel (B), data are the proportion of physicians and dietitians who selected each prespecified response option (HCP Q515). HCP, healthcare professional; N/A, not applicable; PwO, people with obesity; QoL, quality of life.

Physicians and dietitians most frequently recommended that their patients with obesity should be more active (recommended to 76% and 86% of patients, on average) and improve their eating habits (recommended to 67% and 85% of patients, on average); physicians also frequently recommended visiting a dietitian (to 59% of patients, on average; shown in Figure 4). Physicians and dietitians recommended bariatric surgery to only 16% and 20% of their patients with obesity, while prescription weight‐loss medications were recommended to 26% and 11%. Physicians and dietitians believed that being more active (selected by 72% and 92%, respectively) and improving eating habits (selected by 78% and 92%, respectively) were two of the most effective long‐term weight‐management methods; however, compared with physicians, a greater proportion of dietitians thought that behaviour therapy was an effective method (selected by 48% of physicians compared with 80% of dietitians; shown in Figure 4).

In terms of attitudes towards prescription weight‐loss medications, most PwO would rather lose weight themselves than depend on prescription medications; less than half would like their HCP to offer prescription weight‐loss medication (shown in Figure S7). By comparison, fewer HCPs agreed that their patients would rather lose weight themselves than depend on medication, and most physicians agreed that their patients would like them to offer prescription weight‐loss medication. However, most physicians indicated that they were likely to prescribe new prescription weight‐loss medications, and less than a quarter were concerned about associated side effects. Overall, only 6% of PwO who had made a serious weight‐loss attempt were currently trying prescription weight‐loss medications.

In terms of weight‐loss surgery, most PwO would rather lose weight with diet and exercise, and a similar proportion of HCPs would rather motivate their patients to lose weight with diet and exercise (shown in Figure S7). Around two‐thirds of HCPs agreed weight‐loss surgery is more effective than other treatment options, compared with around one‐third of PwO. Despite this, only 5% of PwO had ever had bariatric surgery.

### 3.6 HCP follow‐up

On average, physicians reported scheduling follow‐up appointments to discuss weight with 54% of their patients with obesity, whereas dietitians did so for 90%. However, only 38% of PwO who had discussed weight with an HCP indicated that a weight‐related follow‐up appointment was scheduled after their last visit.

Most physicians and dietitians agreed that one of the first methods of treating obesity should be referring the patient to a dietitian (before prescription medication or bariatric surgery; 83% and 100%) and that physicians should refer patients with obesity to a dietitian earlier once obesity is identified (72% and 96%). For both physicians and dietitians, the most common reason for referring a patient with obesity for specialized obesity management is an indication for bariatric surgery (selected by 81% and 84%). Most dietitians (72%) indicated that PwO are typically under their care for 6 months to a year.

## 4 DISCUSSION

ACTION Switzerland identified perceptions, attitudes, behaviours and potential barriers to effective obesity care among PwO and HCPs (including physicians and dietitians). The results suggest that most PwO in Switzerland underestimate their weight status, which is not surprising, given that approximately half of the PwO surveyed had not discussed their weight with an HCP in the past 5 years. A top reason for HCPs not initiating weight discussions was a perceived lack of motivation to lose weight among PwO; HCPs also appeared to underestimate motivation among PwO. As such, HCP misperceptions may act as a barrier to effective communication. Furthermore, although most respondents agreed that obesity is a chronic disease, the majority of PwO believed they were entirely responsible for their weight loss, and a subset of HCPs (primarily dietitians) did not agree that they have a responsibility to actively contribute to their patients' weight‐loss efforts (i.e. did not select one of the top two response options on the 5‐point Likert scale). These attitudes could prevent PwO from seeking support. Taken together, our results highlight a need for better communication between PwO and HCPs, and greater recognition that obesity is a chronic disease that benefits from early intervention.

In this study, most PwO underestimated their weight class, with the majority considering themselves to have overweight. Similar misperceptions of body size and weight class compared with recognized clinical obesity criteria have been reported in other countries.^8^, ^11^, ^12^, ^15^, ^16^, ^17^, ^18^, ^19^ Failure to recognize one's weight status may cause patients to postpone discussing their weight with HCPs, refuse to accept an obesity diagnosis and/or delay obesity management/treatment, thus limiting intervention efforts. As such, there is a need to better inform PwO about their weight status, the fact that obesity is a disease, possible consequences of obesity and weight‐management strategies. Just over half of PwO (57%) in this study considered obesity to be a chronic disease. Because survey data were pooled, it was not possible to compare answers between PwO who considered obesity to be a chronic disease and those who did not. This type of analysis should be considered in future studies as it would help inform how intervention efforts can be maximized.

Among HCPs, there was a disconnect between their recognition of obesity as a chronic condition and the delayed initiation of conversations about weight. HCPs' top reason for initiating weight‐related conversations was the presence of obesity‐related comorbidities, with many not initiating such conversations due to a perceived lack of motivation to lose weight. However, just over half of PwO who had not discussed weight with their HCP would have liked them to initiate such conversations, and a higher proportion of PwO were motivated to lose weight than was acknowledged by HCPs. HCPs play a central role in introducing discussions about weight, so should be aware of the importance of initiating such discussions earlier after obesity is diagnosed in order to minimize the risk of future complications, which appear to be correlated with the duration of obesity.^20^, ^21^, ^22^, ^23^, ^24^, ^25^ On the other hand, we found that most PwO whose HCP usually initiates weight‐related discussions did not like that their HCP raises the topic, so HCPs should be mindful of patients' feelings when initiating such discussions. Of note, communication between HCPs and patients can be improved through the use of techniques such as motivational interviewing, a patient‐centred method that aims to explore and resolve patients' ambivalence towards change in a non‐judgemental and collaborative discussion style, with demonstrated effectiveness in helping patients to make and maintain changes in their lifestyle.^26^, ^27^

The stigma of obesity plays an important role in weight management. In our study, many respondents believed it is more difficult for PwO to gain employment, have romantic relationships and even make friends. This is not surprising, given that stigmatizing attitudes towards overweight/obesity have been documented in key areas of living, including employment, and negative attitudes have also been reported in healthcare settings.^5^, ^28^ It is therefore interesting that metabolic pathology and the genetic origin of obesity were not among HCPs' most commonly identified barriers to weight loss in the present study, despite most HCPs recognizing obesity as a chronic disease. Of note, we found that a much higher proportion of dietitians believe weight loss is entirely their patients' responsibility, compared with physicians. There remains a need to reduce stigma in healthcare settings through education about the complex causes of obesity, including factors not within an individual's control.

The pervasive nature of stigma throughout society can lead to internalized stigma in PwO. This is a barrier to collaborative treatment as it may lead PwO to assume complete responsibility for weight loss or make them feel uncomfortable seeking support from HCPs.^29^ Indeed, in this study, three‐quarters of PwO believed weight loss is entirely their own responsibility, compared with approximately one‐quarter of physicians. Internalized stigma has also been associated with a range of psychological and physical outcomes, including depression, anxiety, poor quality of life and self‐esteem, and disordered eating,^30^, ^31^ which can lead to further weight gain and increased risk of cardiometabolic complications.^32^, ^33^ Furthermore, internalized stigma at the start of treatment has been associated with poorer weight‐loss outcomes at follow‐up.^34^ As such, baseline levels of internalized stigma should be determined prior to initiating weight‐loss treatment.

Perceptions of weight‐loss interventions varied between PwO and HCPs in this study. Physicians highly regarded prescription weight‐loss medication and bariatric surgery, while PwO tended to favour behavioural interventions that incorporate dietary changes and increased physical activity. Considering this, and the tendency for PwO to believe that weight loss is entirely their responsibility and that they already know how to manage their weight, one could speculate that PwO may have internalized the ‘eat less, move more’ mantra, which should be avoided as it fails to address the complexity of weight management and is unlikely to result in long‐term improvement.^7^, ^35^ Internalized stigma and lack of awareness of the genetic and metabolic components of obesity may result in some PwO viewing medical intervention as a weakness. There is therefore a need for HCPs to inform PwO about the complex nature of obesity and all available treatment options, including lifestyle interventions, behaviour therapy/psychotherapy, pharmacotherapy and bariatric surgery.

This was the first ACTION study to survey dietitians. Compared with physicians, dietitians were more likely to consider behaviour therapy to be an effective long‐term weight‐management method and schedule follow‐up appointments with patients. Increased involvement of registered dietitian nutritionists has been shown to promote improvements in diet‐related clinical outcomes,^36^ indicating that increased collaboration between physicians and dietitians would be beneficial. In an ideal scenario, PwO would receive care from a multidisciplinary network that includes, in addition to their primary care practitioner and a dietitian, an obesity specialist, a physical activity specialist, a psychologist/psychiatrist and a nurse.^27^ In this study, most HCPs agreed that dietitians should be involved early in the treatment process following obesity diagnosis.

Many of the findings from ACTION Switzerland suggest a need to offer HCPs additional educational support. This could help to increase HCPs' awareness of patients' attitudes towards weight management, particularly their motivation to lose weight, which HCPs underestimated in this study. However, as the HCPs surveyed were already aware that obesity is a chronic disease, we believe it would be most beneficial to offer postgraduate obesity seminars focussed on practical recommendations for effective obesity management. These should highlight the need to set realistic weight‐loss targets, i.e. a reduction of 5–10% body weight^27^ (as PwO indicated HCPs recommended a reduction of 21.5%, on average); the importance of scheduling obesity‐related follow‐up appointments (as physicians reported doing so for only 54% of PwO, on average); and the benefits of multidisciplinary treatment and referral for specialized obesity management (as physicians reported recommending behaviour therapy and visiting an obesity specialist to only 22% and 28%, respectively, of PwO, on average). Interestingly, although physicians and dietitians recommended behaviour therapy to only 22% and 39% of PwO (on average), 48% of physicians and 80% of dietitians considered behaviour therapy to be an effective long‐term weight‐management method. We hypothesize that this discrepancy may be due to HCPs perceiving behaviour therapy with a psychologist/psychiatrist as expensive and time‐consuming. This underscores the importance of referring PwO for specialized obesity management, as many obesity specialists in Switzerland include aspects of behaviour therapy in their treatment plans.^27^ However, as noted previously, PwO should ideally receive care from a multidisciplinary network that includes a psychologist/psychiatrist.

Overall, the findings from ACTION Switzerland are broadly in line with those from the ACTION International Observation study,^10^ which suggests that perceptions, attitudes, behaviours and barriers to effective obesity care in Switzerland are similar to those in the wider international community. Both studies highlight the need for improved communication and appropriate weight‐management conversations between HCPs and PwO. Additionally, both suggest there is an opportunity to improve obesity management by offering HCPs additional educational support concerning effective obesity management and promoting a multidisciplinary and multimodal treatment approach. When looking at specific regions, data from ACTION Switzerland were also broadly similar to data from North America,^8^, ^9^ Asia‐Pacific,^12^, ^37^ Denmark and France.^11^, ^38^ However, differences were seen across regions in the proportions of PwO who considered obesity to be a chronic disease. Although the proportions of PwO recognizing obesity as a chronic disease were relatively similar in ACTION Switzerland, North America and France (ACTION Switzerland: 57%; ACTION Canada: 60%; ACTION USA: 65%; ACTION‐FRANCE: 62%),^8^, ^9^, ^38^ more PwO in Asian populations recognized obesity as a chronic disease (ACTION APAC: 68%; ACTION‐China: 77%).^12^, ^37^ In contrast, in Denmark, a lower proportion of PwO recognized obesity as a chronic disease (ACTION‐DK: 49%).^11^ Regional differences in recognizing obesity as a chronic disease could be attributed to cultural nuances and how obesity is viewed in different societies. It should be noted that these comparisons are based on observations only and direct comparisons could not be made. Future research, possibly in different patient groups, is needed to clarify the role of perceptions of (and attitudes towards) obesity as possible obstacles to effective treatment. Additionally, further research is required to explore the efficacy of multidisciplinary treatment programmes.

Strengths of ACTION Switzerland include that this was the first study assessing the perceptions, attitudes and behaviours of PwO in Switzerland. It was also the first to survey PwO, physicians and dietitians, which has allowed us to compare the attitudes, perceptions and behaviours of physicians and dietitians for the first time. Finally, selection bias was mitigated by the stratified sampling and demographic weighting of PwO; this, combined with the recruitment of a large PwO sample, should make it possible to generalize our findings to the broader population of PwO in Switzerland.

Limitations of this study include its cross‐sectional nature and the calculation of BMI based on self‐reported data. Additionally, low response rates were observed, which may have introduced an element of selection bias; however, low response rates are a known limitation of survey‐based studies, particularly those that invite prospective participants via online channels (as in this study), and the response rates observed in this study were in line with expectations for survey‐based studies. Finally, dietitian data were based on a small sample of dietitians, so may not be representative of the broader population of dietitians practising in Switzerland.

In conclusion, ACTION Switzerland compares, for the first time, the perceptions, needs, beliefs and goals of HCPs (including physicians and dietitians) with those of PwO in Switzerland. ACTION Switzerland also builds upon the findings of the ACTION International Observation study by incorporating responses from dietitians (a key HCP demographic in obesity management) and including questions relating to weight stigma. Overall, the data suggest that there is a need to reduce stigma by educating HCPs and raising awareness among PwO that obesity is a chronic disease, and as such, PwO should not assume complete responsibility for weight loss. There is also a need to improve communication between HCPs and PwO, for example, by utilizing behavioural interventions.

## AUTHOR CONTRIBUTIONS

All authors contributed to the design of the study (i.e. by reviewing the surveys) and interpretation of data. Additionally, all authors participated in drafting and revising the article, provided approval to submit the final article and agree to be accountable for all aspects of the publication.

## FUNDING INFORMATION

This study was sponsored by Novo Nordisk. Representatives of the sponsor were involved in designing the study; interpreting the data; and drafting, reviewing and approving the manuscript. The sponsor funded data collection and analysis (by KJT Group) and medical writing assistance. The sponsor also paid the Open Access fee.

## CONFLICT OF INTEREST STATEMENT

During the conduct of the study, the Swiss Obesity Alliance received consultancy fees from Novo Nordisk for the role of its board members (DD, PP, RP, DFT and PAG) and secretary (GF) as members of the ACTION Switzerland Steering Committee; DD, PP, RP, DFT, GF and PAG did not receive payment from the Swiss Obesity Alliance for their role in the ACTION Switzerland Steering Committee. Outside the submitted work, the authors also disclose the following: RP reports grants from the Swiss National Science Foundation; lecture and consulting fees (paid to his institution) from the Falk Foundation, Johnson & Johnson and Viatris; consulting fees (paid to his institution) from Lilly; and fees paid to his institution for participation on an advisory board for Johnson & Johnson. GF is Vice President of the Swiss Association of Professional Organizations in the Healthcare Sector and Secretary of the Swiss Diabetes Foundation and the Professional Association for Obesity in Childhood and Adolescence. GC is an employee of (and shareholder in) Novo Nordisk. PAG reports grants from the Swiss National Science Foundation; consulting fees from Amgen, Boehringer Ingelheim, Lilly, Novo Nordisk and Sanofi; honoraria from Amgen, Lilly and Novo Nordisk; support for attending meetings and travel from Novo Nordisk; and participation on advisory boards for Amgen, Lilly and Novo Nordisk. He is also a committee member of the Swiss Society of Endocrinology.

## Supporting information


**Data S1.** Supporting Information.

## Data Availability

Data will be shared with bona fide researchers submitting a research proposal approved by the independent review board. Individual participant data will be shared in data sets in a de‐identified and anonymized format. Data will be made available after research completion. Information about data access request proposals can be found at novonordisk-trials.com.
