# Supplementary material for: Improving obesity management: Insights from the ACTION Switzerland survey of people with obesity, physicians and dietitians
Source: Clin Obes. 2024 Nov 7;15(2):e12716. doi: 10.1111/cob.12716 (PMC11907096; doi:10.1111/cob.12716)
Supplement: Supplementary file 1 — Data S1. Supporting Information. [file COB-15-e12716-s001.pdf]

Supplementary material

Improving obesity management: Insights from the ACTION Switzerland survey of people with obesity, physicians and dietitians

Dominique Durrer[1]* | Patrick Pasi[2]* | Ralph Peterli[3] | Doris Fischer-Taeschler[4] | Gabriela Fontana[5] | Gionata Cavadini[6] | Philipp A. Gerber[7]

[1]Obesity Centre, Eurobesitas EASO COMs Centre, Vevey, Switzerland

[2]Department of Consultation-Liaison Psychiatry and Psychosomatic Medicine, University Hospital Zürich, University of Zürich, Zürich, Switzerland

[3]Department of Visceral Surgery, Clarunis, University Digestive Health Care Center, St. Clara Hospital and University Hospital Basel, Basel, Switzerland

[4]President, Swiss Obesity Alliance, Baden, Switzerland

[5]Managing Director, Swiss Obesity Alliance, Baden, Switzerland

[6]Clinical, Medical and Regulatory, Novo Nordisk Pharma AG, Zürich, Switzerland

[7]Department of Endocrinology, Diabetology and Clinical Nutrition, University Hospital Zürich (USZ) and University of Zürich (UZH), Zürich, Switzerland

*Co-first authors

Correspondence

Philipp A. Gerber, Department of Endocrinology, Diabetology and Clinical Nutrition, University Hospital Zürich, Ramistrasse 100, 8091 Zürich, Switzerland

Tel: +41 44 255 16 65

Email: Philipp.Gerber@usz.ch

CONTENTS

SUPPLEMENTARY METHODS 3

SUPPLEMENTARY REFERENCES..7

SUPPLEMENTARY TABLES/FIGURES 8

SURVEY INVITE: PwO..23

SURVEY INVITE: HCPs 24

SURVEY QUESTIONS: PwO 25

SURVEY QUESTIONS: HCPs...55

SUPPLEMENTARY METHODS

1 | SURVEY DEVELOPMENT

Two surveys were specifically developed for this cross-sectional study; they were not validated for measurement consistency as they were not developed for repeated use or measuring clinical outcomes. Including the screening form, the surveys had 8 to 9 sections and 59 to 65 closed-ended questions (excluding quality control questions and calculations for variables like age, region and body mass index).

1.1 | Quality control

KJT Group programmed the surveys and completed rigorous quality checks. Each survey path was thoroughly tested (in a variety of software packages for web browsers and mobile devices) to ensure that all text was accurate and the programmed pathways were functioning as intended.

A set of randomly generated ‘dummy’ data that filled all possible pathways/quotas was run, and a programmatic check was conducted to test the validity of the survey. This tested whether each question contained the correct number of responses, each response was coded accurately, questions were answered by the intended respondents and all variables derived from calculations or algorithms were accurate. Data check edits were then written and approved prior to the ‘soft launch’ (i.e., fielding with a sample designed to recruit 10% of the quota). Data from the soft launch (and subsequently the full launch and the final data set) were checked using the data check edits to ensure programming accuracy.

Data were also monitored at regular intervals, for example, when 10%, 25%, 50% and 75% of data had been collected. In-field data checks were conducted to identify and exclude survey responses that were suspected to be of poor quality (based on inappropriate responses

to quality control questions and unusually short survey completion times, i.e., <10 minutes for surveys that were estimated to require 25 minutes).

2 | PROCEDURES

2.1 | Recruitment

Prospective participants were identified from online panel companies and recruited via email. For dietitians, this was supplemented with custom telephone recruitment (conducted by the online panel company).

The general population sample was stratified to match demographic targets for the general population in Switzerland (age, sex, income, education and language spoken). This ensured that the qualifying sample was largely representative of the population with obesity in Switzerland and minimized the need for demographic weighting. The demographic targets used were from government agencies or recent census data, when available, or peer-reviewed data sources.

The stratified general population sample was then screened to identify people with obesity (PwO). Potential respondents were not directly asked if they had obesity; their body mass index was calculated using self-reported height and weight data, and only those with a body mass index of ≥30 kg/m^2 were able to proceed to the full survey.

2.2 | Data collection

The online surveys were hosted by KJT Group on a secure website. To prevent unauthorized access to the surveys, each participant who was invited to take part in the study was sent a unique survey link; the survey available at this link could only be completed once. Respondents could suspend participation at any time, for any reason, and were able to pause the survey and complete it later.

Measures were taken to protect individual identification and personal information. All data were stored on secure servers. Only necessary information was collected; names or other identifying information were collected to compensate respondents for their time and adhere to adverse event reporting requirements, while demographic data were collected to ensure sample representativeness and for potential use in subgroup analyses. Personal information that could be used to identify respondents was excluded before the encrypted data from the study were transferred to Novo Nordisk.

In this cross-sectional, online survey study, there were no free-text response fields or questions relating to specific medicinal products; therefore, there were no opportunities for respondents to report adverse events or other safety information during survey completion. However, if KJT Group were made aware of adverse events or other safety information relating to Novo Nordisk products during the custom telephone recruitment of dietitians, the relevant information was collected and reported to Novo Nordisk.

2.3 | Compensation

Respondents were compensated for their time. PwO were provided with panel credit from the online panel company and compensation for healthcare professionals was paid at fair market value for each specialty type.

3 | SAMPLE SIZE

Sample sizes were selected to balance statistical power, recruitment feasibility and cost; they were based on usual acceptance of a smallest subsample and previous experience from the Awareness, Care, and Treatment In Obesity maNagement (ACTION) studies conducted in the US and Canada and the global ACTION International Observation study.[1–3] For PwO and physicians, respectively, sample sizes were designed to achieve a 3.1% or 8.8% margin of error (calculated from a standard normal [Z-] distribution, with z=1.96 or approximately 95%

confidence), around a proportion estimate of 50%. For dietitians, the sample size was selected to allow for qualitative descriptive comparisons with physicians.

4 | ANALYSIS

For 5-point Likert scales, some response options were merged. For example, for the scale assessing the impact of health conditions (Supplementary material survey questions: PwO Q405), responses of 4 or 5 (where 1 meant ‘very little impact’ and 5 meant ‘an extreme impact’) were coded as ‘extreme impact’ and are reported as such. Similarly, for scales assessing agreement (e.g., PwO Q640), responses of 4 or 5 (where 1 meant ‘do not agree at all’ and 5 meant ‘completely agree’) were coded as ‘agree’.

Weights were applied to data collected from PwO to mitigate selection bias and increase the generalizability of results. The weights were calculated using a raking technique (i.e., an iterative proportional fitting technique used when population distribution is known) in order to achieve the nearest possible sample and target balance; to avoid extreme design effects, individual respondent weights were capped at 0.5 and 5.0. The final sample, which included those who were not eligible for the study, was weighted on key demographics (sex, age, language region, education and household income) using general population targets from government and other public data. The weight for each respondent was adjusted until the sample distribution aligned with the general population targets for these demographic variables.

SUPPLEMENTARY REFERENCES

1. Kaplan LM, Golden A, Jinnett K, et al. Perceptions of barriers to effective obesity care: results from the national ACTION study. *Obesity (Silver Spring)*. 2018;26(1):61-69.
2. Sharma AM, Bélanger A, Carson V, et al. Perceptions of barriers to effective obesity management in Canada: results from the ACTION study. *Clin Obes*. 2019;9(5):e12329.
3. Caterson ID, Alfadda AA, Auerbach P, et al. Gaps to bridge: misalignment between perception, reality and actions in obesity. *Diabetes Obes Metab*. 2019;21(8):1914-1924.

SUPPLEMENTARY TABLES/FIGURES

TABLE S1 Diagnosed comorbidities.

	PwO
**Full sample, N**	1002
**Comorbidity, %**	
Hypertension	**35**
Depression/anxiety	**21**
High cholesterol	**17**
Stomach or intestinal problems	**12**
Type 2 diabetes	**13**
Obstructive sleep apnea	**10**
Osteoarthritis	**9**
Eating disorder	**7**
Cardiovascular diseases	**10**
Metabolic syndrome	**5**
Pre-diabetes	**5**
Cancer	**4**
Liver disease	**4**
PCOS	**2**
Infertility	**2**
Other	**14**
None of these	**29**

PCOS, polycystic ovary syndrome; PwO, people with obesity.

TABLE S2 Physician specialty.

	Physicians
**Full sample, *N***	125
**Specialty, *n* (%)**	
General internal medicine (with focus on primary care)	55 (44)
Endocrinology/diabetology	23 (18)
General practice physician	19 (15)
General internal medicine (with focus on diabetes and/or obesity)	17 (14)
Obstetrics and gynaecology	8 (6)
Bariatric surgery	2 (2)
Family practice physician	1 (1)
Psychiatry	0

FIGURE S1 Sample disposition for PwO (A) and HCPs (B).

(A) PwO

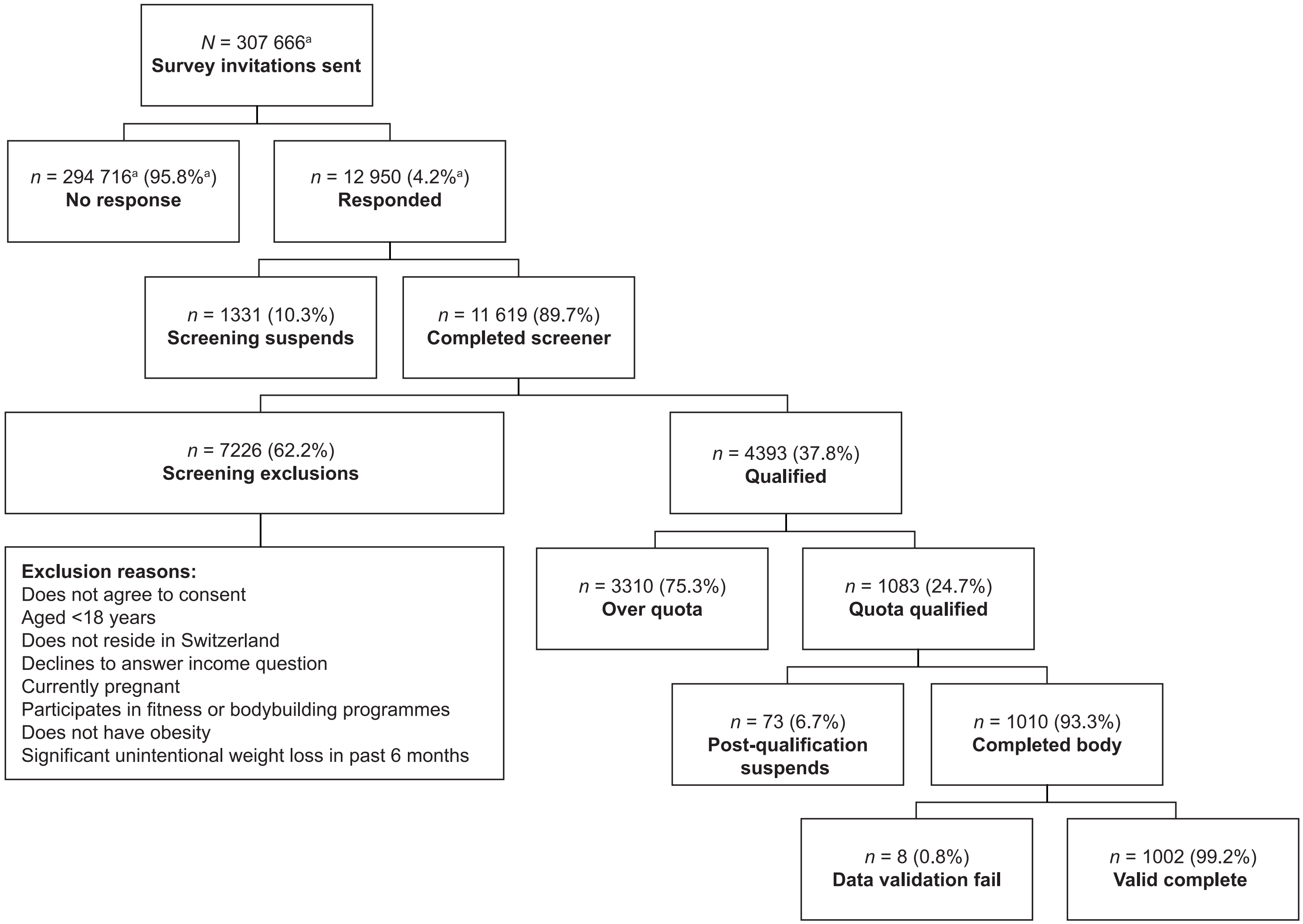

(B) HCPs

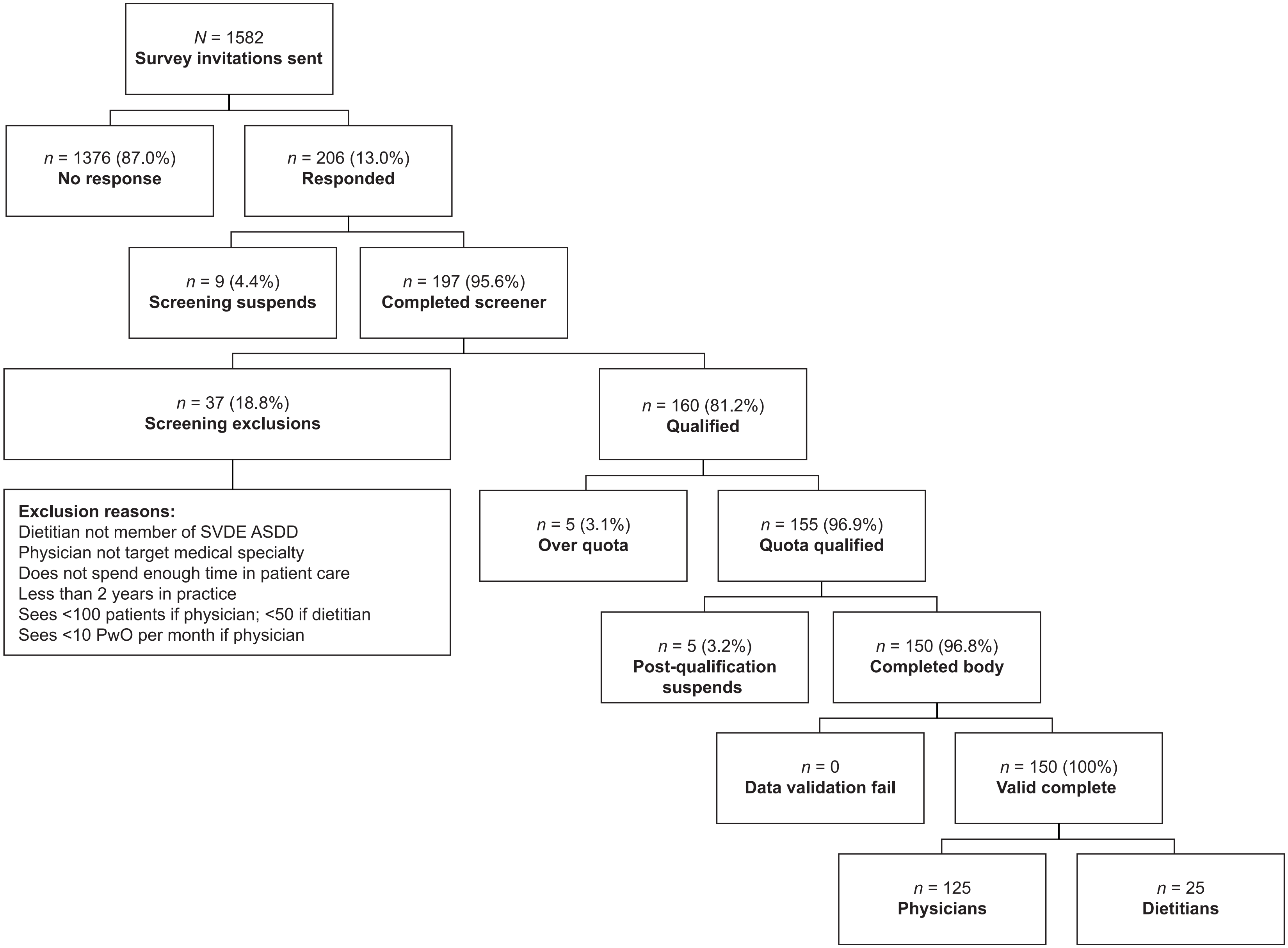
N = 1582
Survey invitations sent
n = 1376 (87.0%)
No response
n = 206 (13.0%)
Responded
n = 9 (4.4%)
Screening suspends
n = 197 (95.6%)
Completed screener
n = 37 (18.8%)
Screening exclusions
Exclusion reasons:
Dietitian not member of SVDE ASDD
Physician not target medical specialty
Does not spend enough time in patient care
Less than 2 years in practice
Sees <100 patients if physician; <50 if dietitian
Sees <10 PwO per month if physician
n = 160 (81.2%)
Qualified
n = 5 (3.1%)
Over quota
n = 155 (96.9%)
Quota qualified
n = 5 (3.2%)
Post-qualification suspends
n = 150 (96.8%)
Completed body
n = 0
Data validation fail
n = 150 (100%)
Valid complete
n = 125
Physicians
n = 25
Dietitians

Responded: indicates that the respondent clicked the link on the survey invitation; the response rate reports the proportion who responded relative to the number of survey invites sent. Screening suspends: respondent did not complete the qualification section of the survey (i.e., screening drop-out). Over quota: respondent qualified but was not allowed to continue to the survey because sufficient data had already been collected from other respondents who matched the respondent's qualification criteria. Post-qualification suspends: respondent qualified for the survey but did not complete the main survey (i.e., survey drop-out). Data validation fail: respondent failed data validation checks (e.g., for entering incorrect responses to data validity questions, 'straight-lining' rating scale questions or having an extremely short completion time); data were excluded from the final data set. HCP, healthcare professional; PwO, people with obesity; SVDE ASDD, Swiss Association of Dietitians. [a]KJT Group worked with various partners to send survey invites to PwO. It was not possible to determine the number of invites sent to PwO by one major partner, therefore the total number of survey invites sent to PwO (across all partners) and the overall response rate could not be calculated. Panel (A) reports the estimated total number of invites sent and the estimated overall response rate for PwO. Estimates assume the major partner with missing data achieved the same response rate as the other major partner.

FIGURE S2 Attitudes towards obesity and treatment.

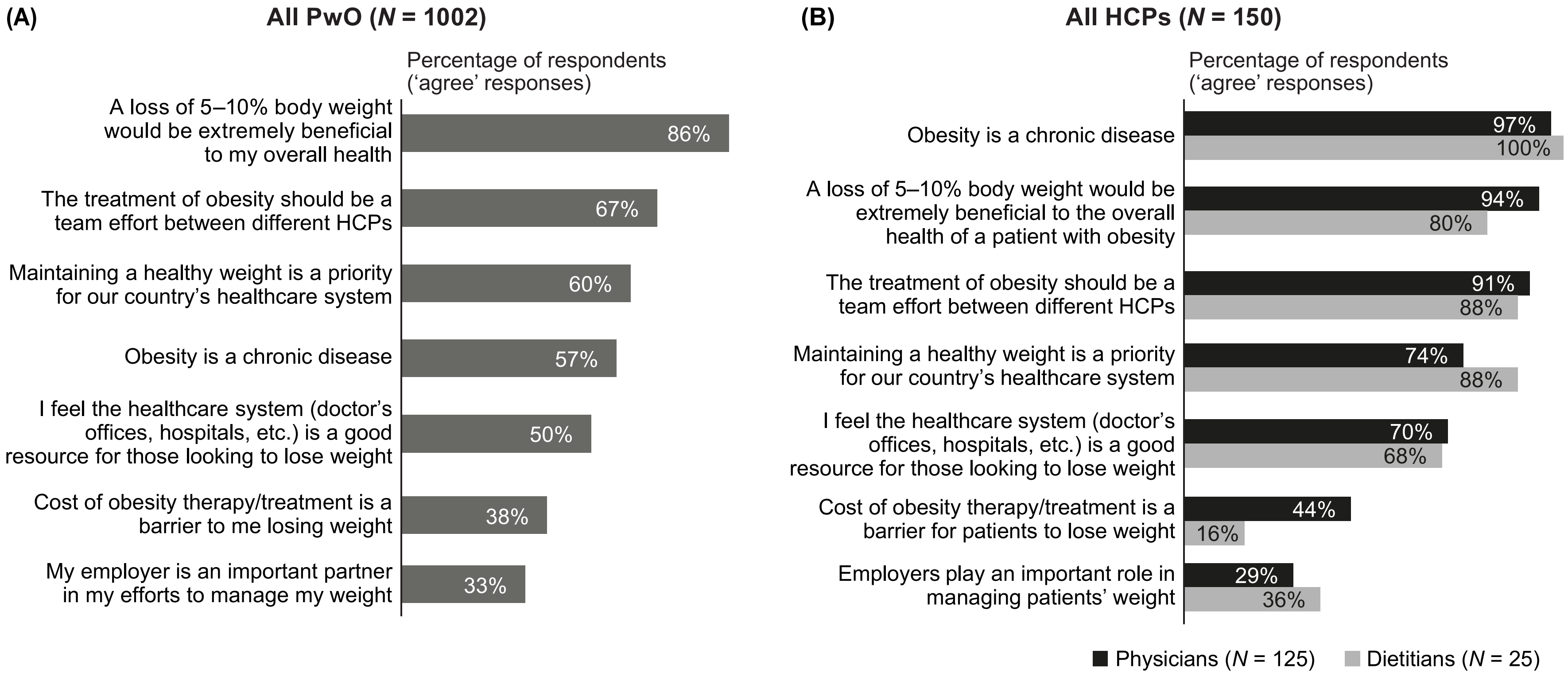

Proportion of PwO, physicians and dietitians who agreed with each statement, i.e., selected one of the top two response options from a 5-point Likert scale where 1 meant 'do not agree at all' and 5 meant 'completely agree' (PwO Q640; HCP Q650). HCP, healthcare professional; PwO, people with obesity.

FIGURE S3 PwO feelings after their most recent weight discussion with their HCP.

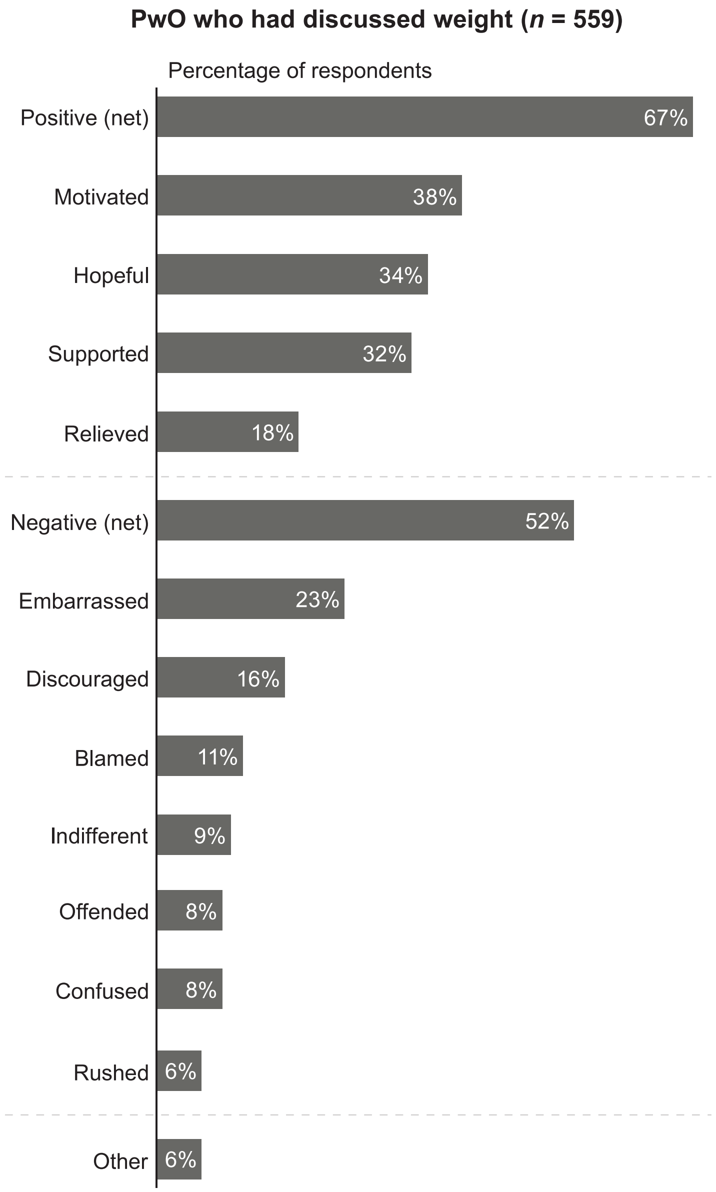

Proportion of PwO who had discussed weight with their HCP in the past 5 years who selected each prespecified response option (PwO Q710). The net positive category reflects the proportion who selected at least one positive feeling (i.e., motivated, hopeful, supported and/or relieved); the net negative category reflects the proportion who selected at least one negative feeling (i.e., embarrassed, discouraged, blamed, indifferent, offended, confused and/or rushed). HCP, healthcare professional; PwO, people with obesity.

FIGURE S4 PwO weight loss in the past 3 years.

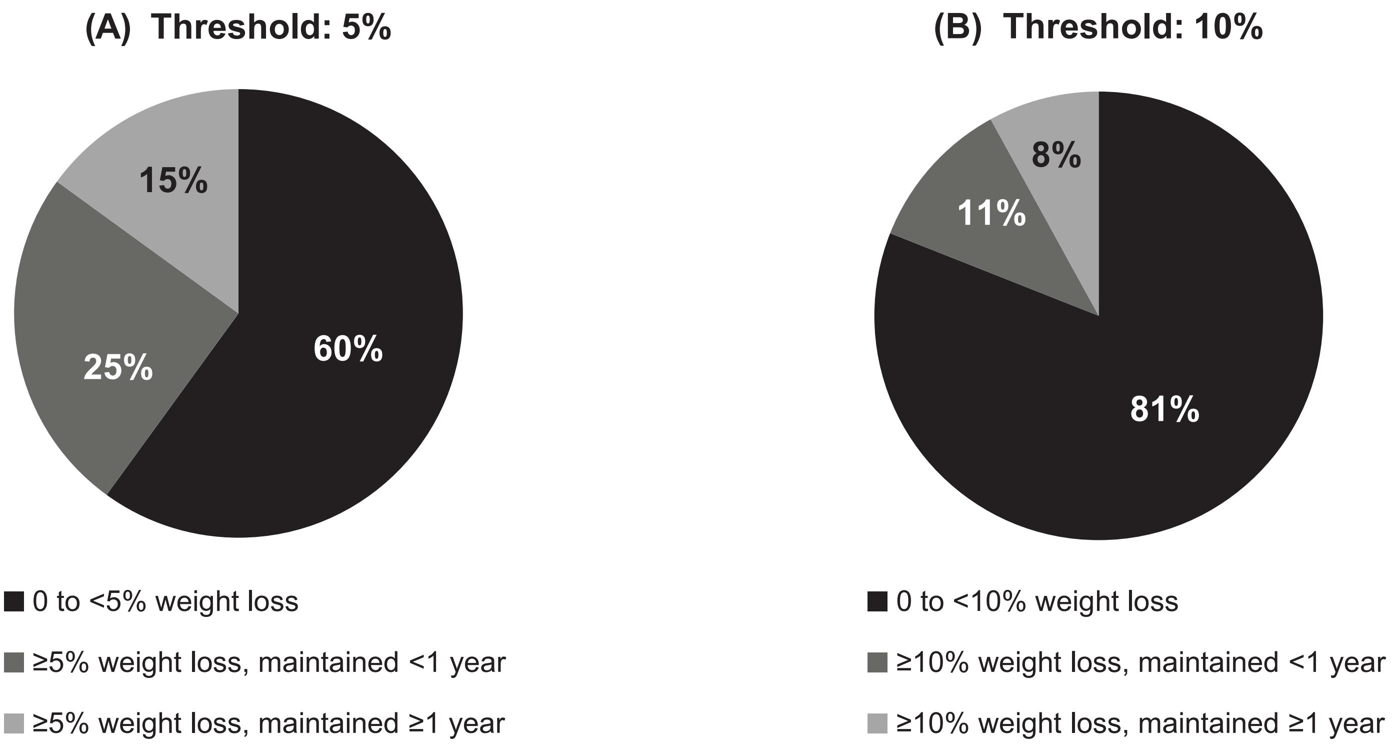

Proportion of PwO (both panels: N = 1002) who: lost <5% or <10% of their body weight; lost ≥5% or ≥10% of their body weight and maintained the weight loss for <1 year; and lost ≥5% or ≥10% of their body weight and maintained the weight loss for ≥1 year. Calculated based on responses to PwO S15, S16 and S19. PwO, people with obesity.

FIGURE S5 Weight-loss motivators according to PwO (A) and HCPs (B).

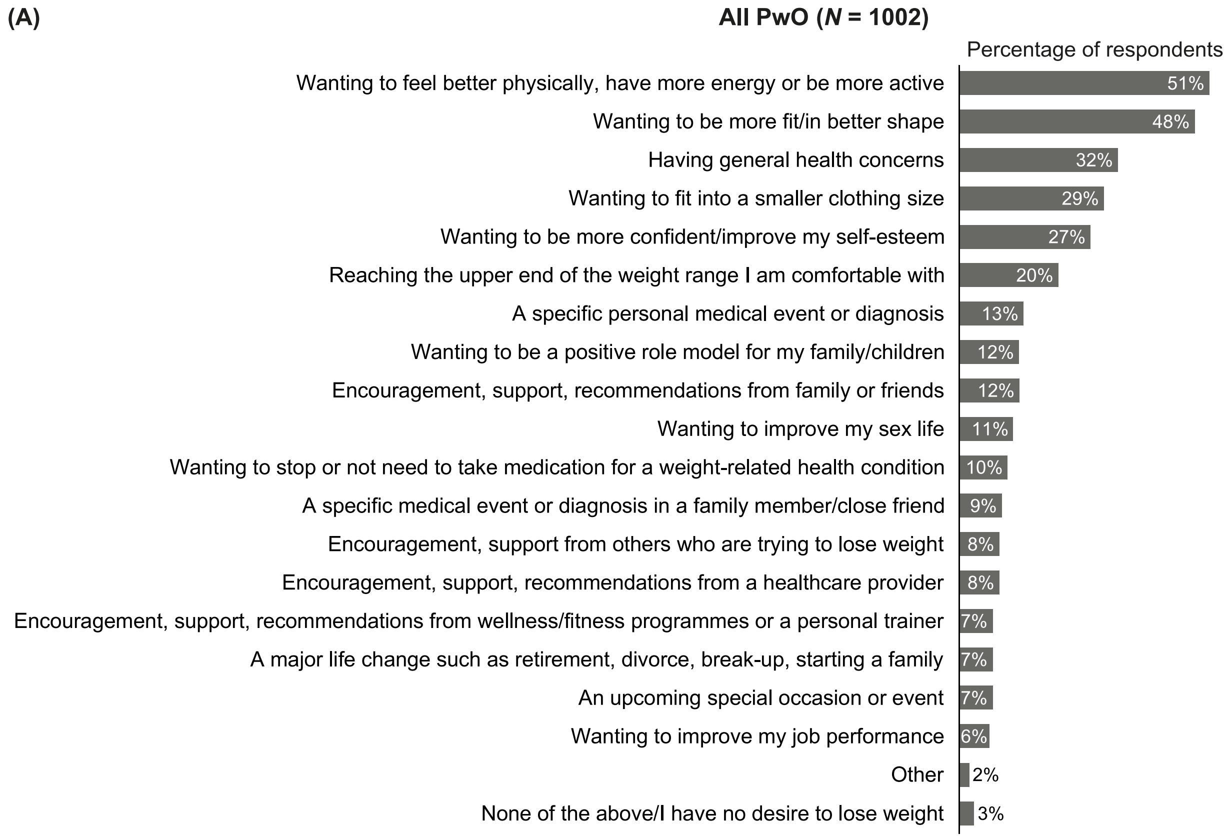

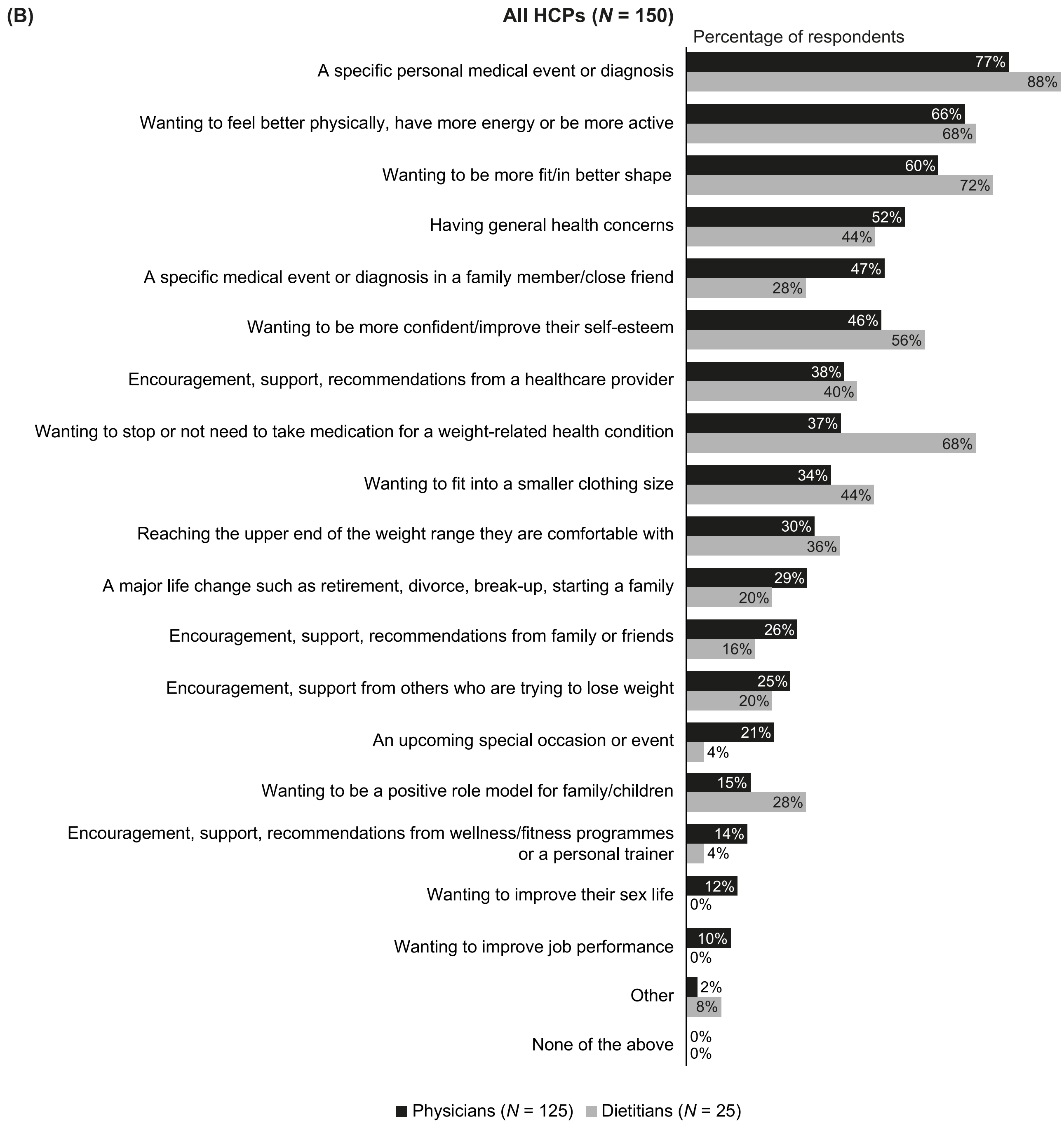

Proportion of PwO, physicians and dietitians who selected each prespecified response option when asked about weight-loss motivators (PwO Q203; HCP Q225). HCP, healthcare professional; PwO, people with obesity.

FIGURE S6 Weight-loss barriers according to PwO (A) and HCPs (B).

(A)

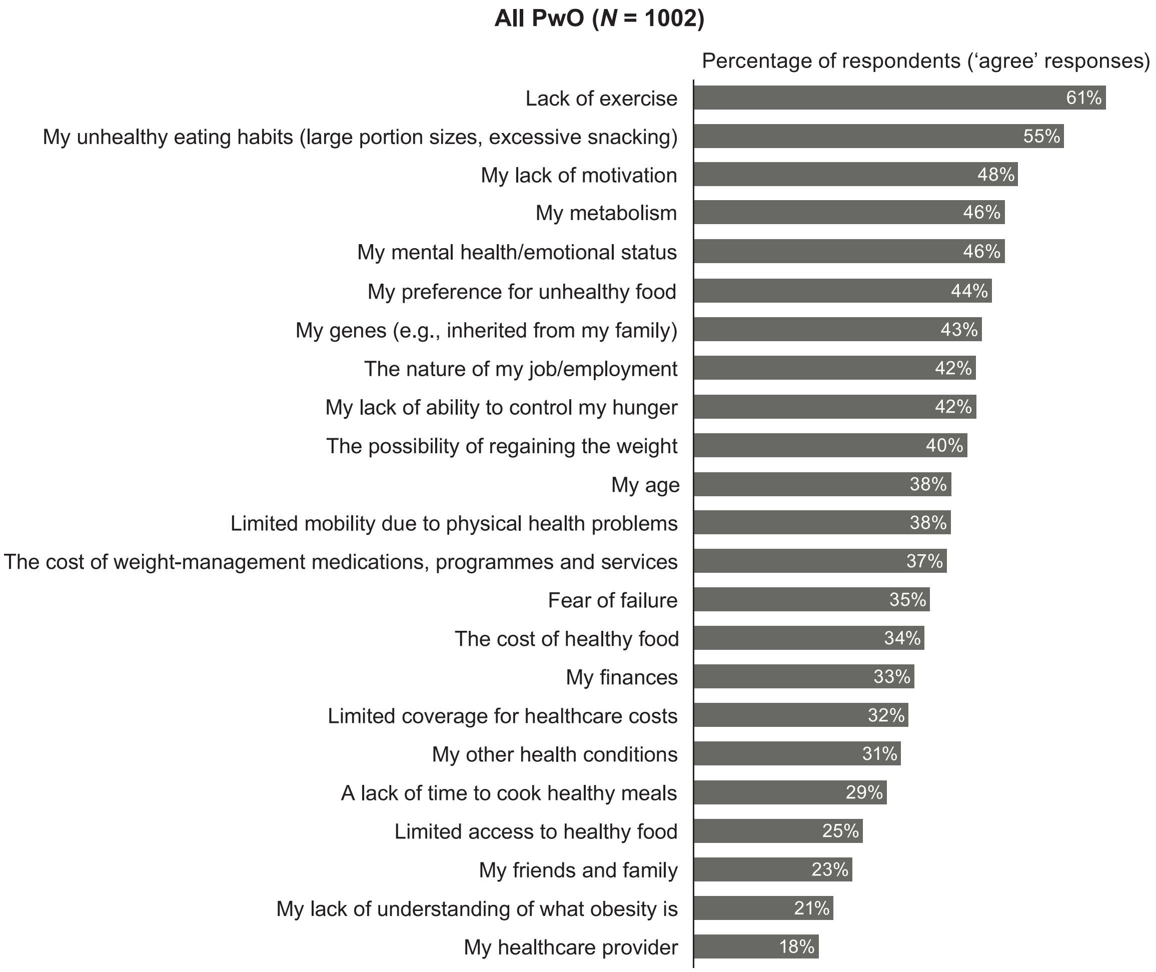

(B)

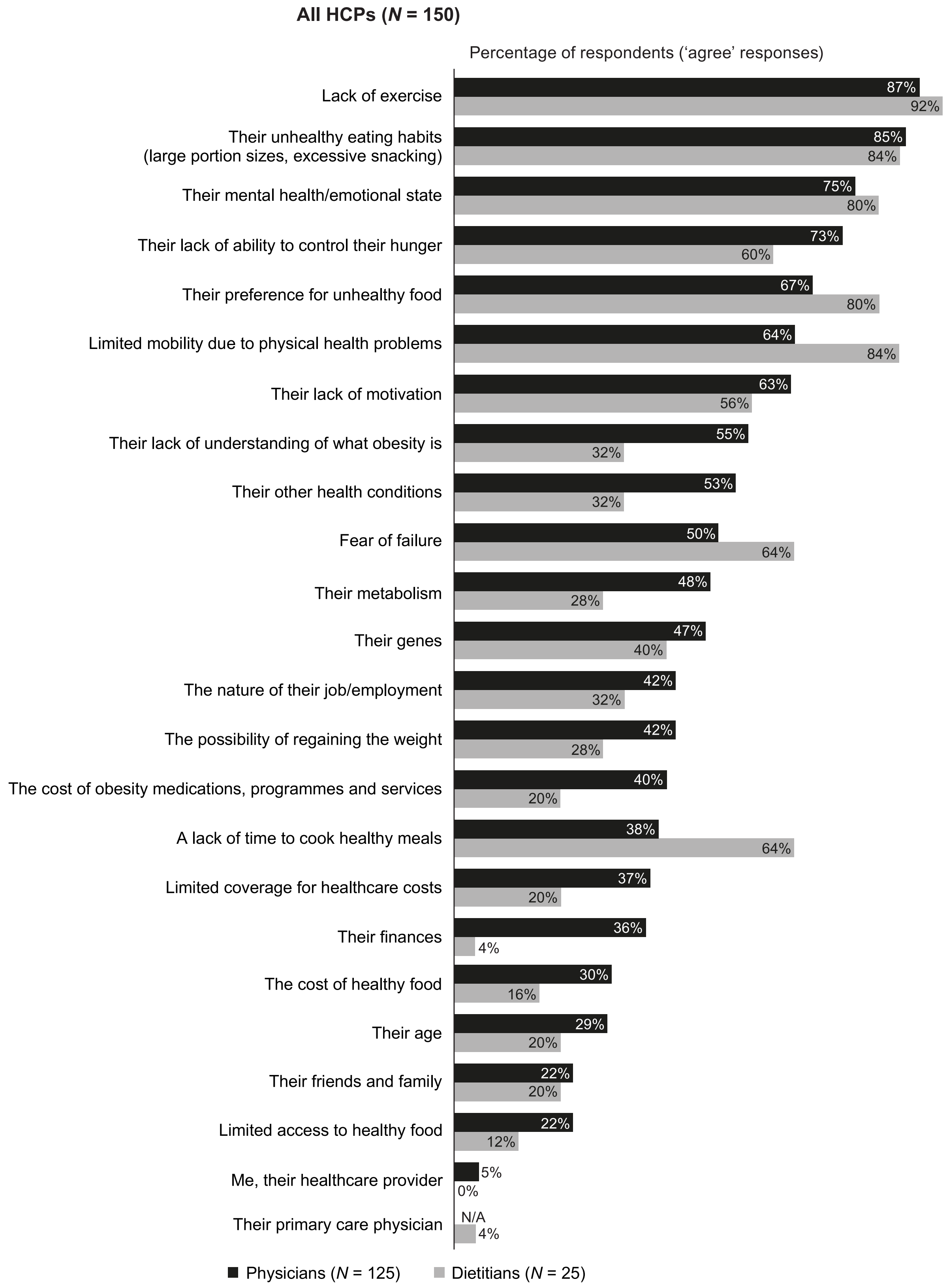

All HCPs (N = 150)
Percentage of respondents (‘agree’ responses)
Lack of exercise 87% 92%
Their unhealthy eating habits (large portion sizes, excessive snacking) 85% 84%
Their mental health/emotional state 75% 80%
Their lack of ability to control their hunger 73% 60%
Their preference for unhealthy food 67% 80%
Limited mobility due to physical health problems 64% 84%
Their lack of motivation 63% 56%
Their lack of understanding of what obesity is 55% 32%
Their other health conditions 53% 32%
Fear of failure 50% 64%
Their metabolism 48% 28%
Their genes 47% 40%
The nature of their job/employment 42% 32%
The possibility of regaining the weight 42% 28%
The cost of obesity medications, programmes and services 40% 20%
A lack of time to cook healthy meals 38% 64%
Limited coverage for healthcare costs 37% 20%
Their finances 36% 4%
The cost of healthy food 30% 16%
Their age 29% 20%
Their friends and family 22% 20%
Limited access to healthy food 22% 12%
Me, their healthcare provider 5% 0%
Their primary care physician N/A 4%
Physicians (N = 125)
Dietitians (N = 25)

Proportion of PwO, physicians and dietitians who agreed that each prespecified response option is a barrier to losing weight, i.e., selected one of the top two response options from a 5-point Likert scale where 1 meant ‘do not agree at all’ and 5 meant ‘completely agree’ (PwO Q507; HCP Q507). HCP, healthcare professional; N/A, not applicable; PwO, people with obesity.

FIGURE S7 Attitudes towards prescription weight-loss methods and surgery among PwO (A) and HCPs (B).

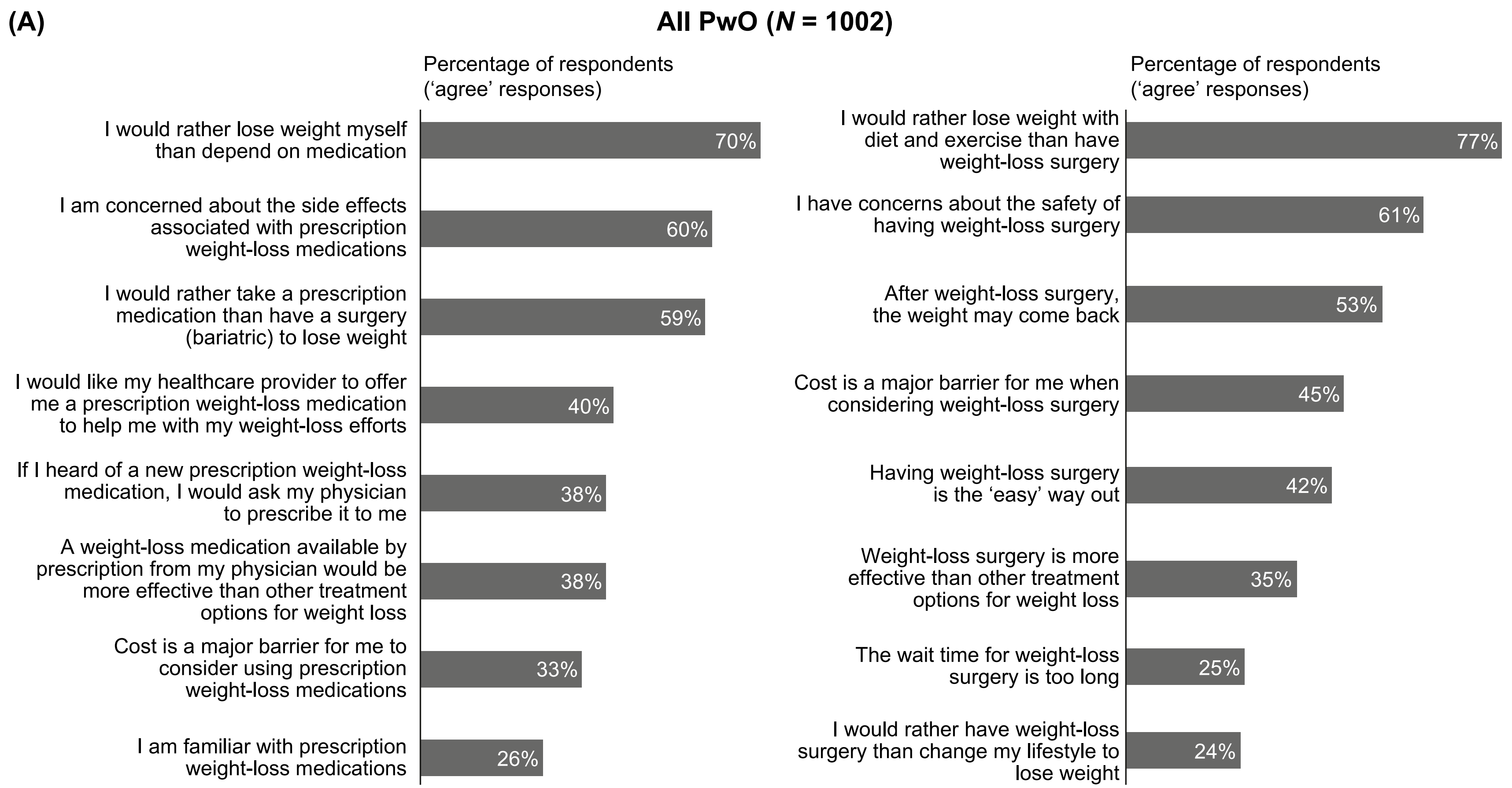

(B)

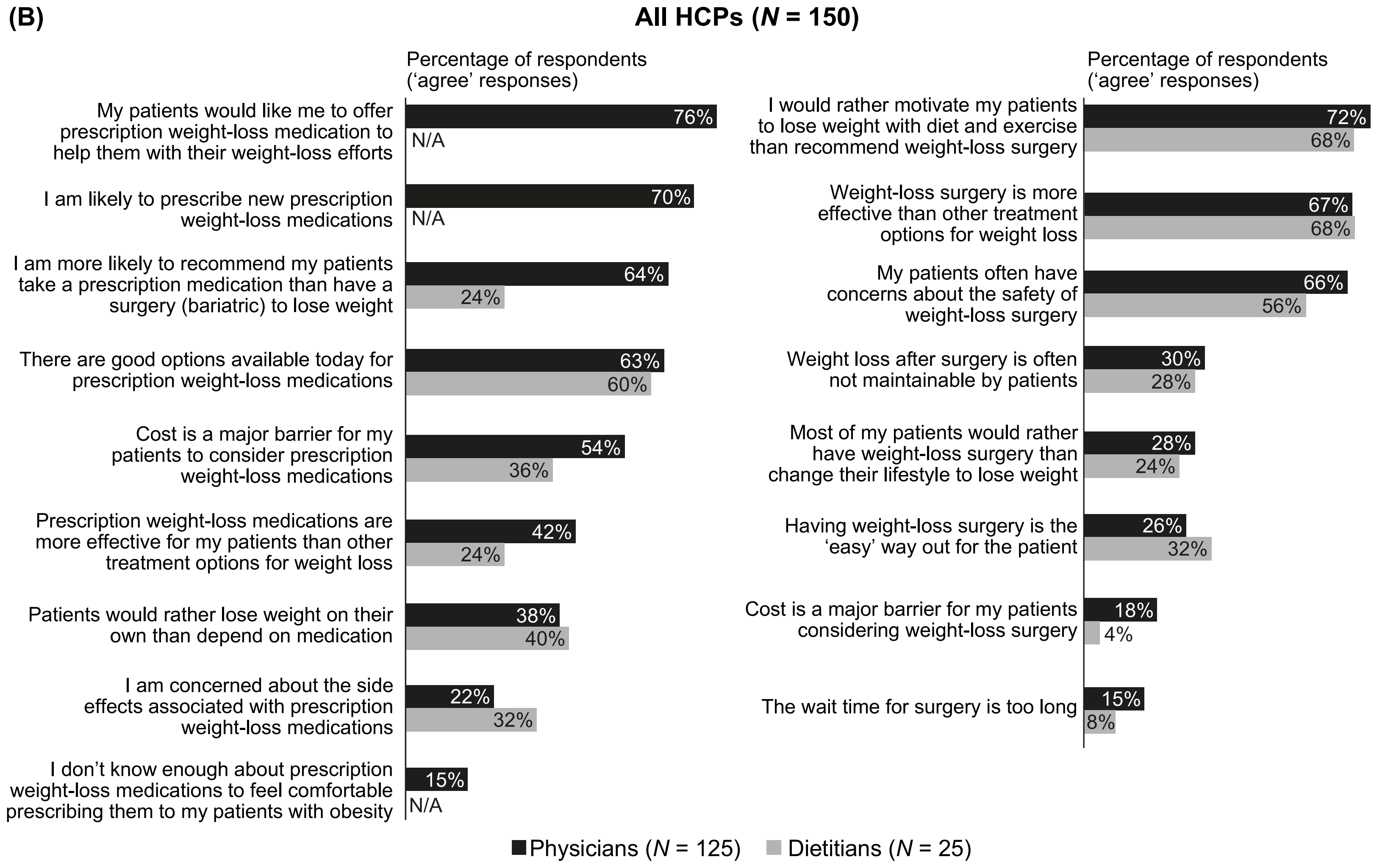

Proportion of PwO, physicians and dietitians who agreed with each statement, i.e., selected one of the top two response options from a 5-point Likert scale where 1 meant 'do not agree at all' and 5 meant 'completely agree' (PwO Q520 and Q525; HCP Q521 and Q525). HCP, healthcare professional; N/A, not applicable; PwO, people with obesity.

SURVEY INVITE: PwO

Email subject line:

Share Your Views – Important Healthcare Survey Opportunity

Email text:

A new survey is waiting for you.

Theme: Home & Family

Bonus: 200 Opinion Points

Survey Length: 25 minutes

Details: Your opinion can help shape the future of your favorite brands and products. Click the link below, find a poll, give your opinion and earn Opinion Points:

XXX

We value your opinions!

SURVEY INVITE: HCPs

Email subject line:

Let us know what you think - an important healthcare survey!

Email text:

Thank you very much for your valuable participation in the online survey on Obesity.

The survey will take around 25 minutes and you will receive compensation of CHF XXX by bank transfer for your participation in this survey.

Please send us your IBAN, postal code and city (from the account holder) no later than when you have completed the survey.

To take part in the survey, please start your personal link:

XXX

(if you have trouble clicking the link, please copy the whole link and paste it into your browser.)

Please remember that there are no right or wrong answers and that we are only interested in your opinion.

We thank you in advance for your cooperation.

SURVEY QUESTIONS: PwO

KJT Group, Inc.
6 East St
Honeoye Falls, NY, 14472

Highlighting color key:
Question asked in ACTION IO
Question asked in ACTION IO, modified for Switzerland
New Question, not asked in ACTION IO

Awareness, Care & Treatment In Obesity MaNagement - ACTION Switzerland - PwO Questionnaire

KJT Group Contacts:

[Name redacted]
Email: [redacted]
Phone: [redacted]

[Name redacted]
Email: [redacted]
Phone: [redacted]

[Name redacted]
Email: [redacted]
Phone: [redacted]

[Name redacted]
Email: [redacted]
Phone: [redacted]

[Name redacted]
Email: [redacted]
Phone: [redacted]

Recruitment:
Target completed interviews: n=1,000

Screening Criteria:

- Age 18+
- Current BMI 30+ calculated based on self-reported height and weight
- Not currently pregnant
- Does not participate in intense fitness or body building programs
- Targeting based on age, gender, education, region

- Respondents will be further classified according to the following:
    - **Maintaining weight loss**: Lost at least 10% of body weight in the past 3 years and has kept weight off for at least a year
    - **Committed to Action**: Intend to take action to lose weight or committed to/enrolled in a plan to lose weight

Data Validation Questions:

Q435, Q500_11, Q640_8

Length of Survey (Target 25-minutes)

SECTION S: SCREENER

ALL RESPONDENTS
S0. Thank you for taking the time to participate in this study. For your convenience we are offering this survey in multiple languages. Please select the language that you are most familiar with.

1. English (United Kingdom)
2. French
3. German
4. Italian

ALL RESPONDENTS
S1 Thank you for your interest in this survey on healthcare issues! For better legibility we abstain from consistently writing all possible gender forms throughout the survey. However, please know that all genders are equally considered.

Before starting, KJT Group requires you to read the following information:

- KJT Group is a **global market research company** requesting your consent to participate in this research survey. KJT Group is the data controller
- KJT's legal basis for the processing of your personal data is your consent
- Your participation is fully voluntary; you can choose to stop at any time and you can, after completion of the questionnaire, withdraw your consent at any time by writing to [KJT email redacted] without any penalty or loss of benefits to which you are otherwise entitled.
- The survey complies with all Swiss and European data protection regulations.
- The purpose of the survey is to help the sponsor (Novo Nordisk) understand the healthcare experiences of patients
- The risk of the research is to your privacy. Your responses will be kept **completely confidential** and will never be connected with your name. Your alternative is to not participate in the survey.
- You have the **right to see and get a copy of your data, amend your data, or erase your data** at any time.
- We expect, on average, it will take about **25 minutes** to complete this survey.
- Your responses will be aggregated with other responses in KJT Group's research report and will be made publicly available in a peer-reviewed scientific journal publication once the study has finished. However, **your name will never be included in the report, publication, or identified to the sponsor**.
- Your responses will be transferred to and **stored on secure servers in the United States**..
- We will **destroy your personally identifiable information within 12 months of data collection**; however, we will maintain a permanent record of this consent.

- If you do not qualify for the study, your personal data will be stored electronically by KJT Group and erased no later than 12 months after the end of data collection.
- All information from this study will be stored for at least 5 years after the Study Report is made, or according to local requirements. The study report contains the full results of the study.
- Your **participation is voluntary,** and you may choose to stop participating at any time (withdraw consent) without any penalty or loss of benefits to which you are otherwise entitled.
- If you qualify and complete this survey, you will receive online panel credit/the honoraria listed in your invitation. There are no costs to you for your participation.
- Your personal information is protected by the data protection law as applicable in your country.
- KJT will make sure that the information we ask about you cannot be looked at by people who are not authorized to do so.

You may contact [KJT email redacted] with questions or concerns, or if you would like to follow-up on these points or need additional support.

Do you agree to these terms and want to continue with the survey?

1. Yes [CONTINUE]
2. No [NOT QUALIFIED, CONTINUE]

ALL RESPONDENTS
S2. Thank you. This survey is focused on your personal experiences, and we very much appreciate your honesty in responding. To begin, we would like to gather some basic information to be used for categorization purposes.

In what year were you born? Please enter as a four-digit number, e.g., 1963.

[RANGE: 1890-2020]
|_|_|_|_|

ALL RESPONDENTS
S3 HIDDEN COMPUTE FOR AGE

[IF AGE 18 OR OLDER (S3>17) ASK S4. ELSE, NOT QUALIFIED, CONTINUE]

AGE 18 OR OLDER (S3>17)
S4. Which gender do you identify with?

1. Male
2. Female
3. Other

AGE 18 OR OLDER (S3>17)
S5. In what canton is your primary residence located?

1) Aargau

2) Appenzell Ausserrhoden
3) Appenzell Innerrhoden
4) Basel-Landschaft
5) Basel-Stadt
6) Bern - French speaking region
7) Bern - German speaking region
8) Fribourg - French speaking region
9) Fribourg - German speaking region
10) Genève
11) Glarus
12) Graubünden - German or Romansh speaking region
13) Graubünden - Italian speaking region
14) Jura
15) Luzern
16) Neuchâtel
17) Nidwalden
18) Obwalden
19) Schaffhausen
20) Schwyz
21) Solothurn
22) St. Gallen
23) Thurgau
24) Ticino
25) Uri
26) Valais - French speaking region
27) Valais - German speaking region
28) Vaud
29) Zug
30) Zurich

99. I do not reside in Switzerland [ANCHOR] [TERMINATE]

HIDDEN VARIABLE:
S5A [REGIONAL RECODES]

1. GERMAN
   [S5/1-5,7,9,11,12,15,17-23,25,27,29,30]
2. FRENCH
   [S5/6,8,10,14,16,26,28]
3. ITALIAN
   [S5/13,24]

AGE 18 OR OLDER (S3>17)
S6. In 2020, what was your household's total yearly income <u>before</u> taxes?

Please remember that your individual information will never be shared. These questions are only used to ensure a representative mix of respondents is achieved.

1. 26,000 Swiss francs or less
2. 26,001 – 52,000 Swiss francs
3. 52,001 - 78,000 Swiss francs
4. 78,001 – 104,000 Swiss francs
5. 104,001 or more Swiss francs
99. Decline to answer [TERMINATE, NOT QUALIFIED]

AGE 18 OR OLDER (S3>17)
S7. What is the highest level of education you have completed?

1. Compulsory education
2. Vocational school
3. Upper secondary specialized school
4. Baccalaureate school
5. College of higher education
6. University of applied sciences or university of teacher education
7. University or Federal Institute of Technology

HIDDEN VARIABLE:
S7A [EDUCATION RECODES]

1. BELOW UPPER SECONDARY (S7r1)
2. UPPER SECONDARY (S7r2-4)
3. TERTIARY (Sr5-7)

ALL RESPONDENTS
S8. DEMOGRAPHIC TARGETS (%) – HIDDEN QUESTION
DEMOGRAPHIC TARGETS (%)

**Category**	**Targets**
Region	
Region code 1 – German	63%
Region code 2 – French	23%
Region code 3 – Italian	14%
Gender	
Male	49.6%
Female	50.4%
Other	1%
Age	
18-24 years	9%
25-34 years	16%
35-44 years	16%
45-54 years	18%
55-64 years	17%
65 and over	24%
Education	
Education code 1 Below upper secondary	5%

Education code 2 Upper Secondary	48%
Education code 3 Tertiary	47%
Income	
26,000 Swiss francs or less	5%
26,001 – 52,000 Swiss francs	6%
52,001 - 78,000 Swiss francs	35%
78,001 – 104,000 Swiss francs	27%
104,001 or more Swiss francs	27%

[IF FEMALE OR OTHER (S4/2-3) ASK S9. ELSE CONTINUE]

FEMALE OR OTHER (S4/2-3)
S9. Are you currently pregnant?

1. Yes TERMINATE
2. No CONTINUE

AGE 18 OR OLDER (S3>17)
S10. Do you participate in intense fitness or body building programs and consider yourself extremely fit?

1. Yes TERMINATE
2. No CONTINUE

AGE 18 OR OLDER (S3>17)
S11. What is your height?

Your best estimate will do.

Please enter your height in centimeters.

Centimeters: |_|_| [RANGE 75-250]

AGE 18 OR OLDER (S3>17)
S12. What is your current weight?

Please be as exact as possible.

[RANGE 25-300
Kilograms: |_|_|_|

S13. HIDDEN QUESTION FOR BMI CALCULATION

S11 convert to m, where 1 m = 100 cm
BMI = S12 kg / [S11 m ^2]

Example: S12 Weight = 68 kg, S11 Height = 165 cm (1.65 m)

Calculation: 68 ÷ $(1.65)^2$ = 24.98

S14. HIDDEN QUESTION FOR WEIGHT CLASSIFICATION

1. Underweight (S13 < 18.5)
2. Normal Range (S13 ≥ 18.5 AND S13 < 25)
3. Overweight (S13 ≥ 25 AND S13 < 30)
4. Obesity Class I (S13 ≥ 30 AND S13 < 35)
5. Obesity Class II (S13 ≥ 35 AND S13 < 40)
6. Obesity Class III (S13 ≥ 40)

[IF CURRENT BMI 30+ (S13 ≥ 30) ASK S15. ELSE, TERMINATE.]

BMI 30+ (S13 ≥ 30)
S15. What is the **least** you have weighed in the past 3 years?
Your best estimate will do.

[RANGE 0 to S12 - 1]
Kilograms: |_|_|_|

99. [ ] I currently weigh the least I've weighed in the past 3 years [EXCLUSIVE]

[IF CURRENTLY MIN WEIGHT (S15/99) AUTO FILL S15 WITH S12]

BMI 30+ (S13 ≥ 30)
S16. What is the most you have weighed in the past 3 years?

[IF FEMALE OR OTHER (S4/2-3) show: "Please do not consider pregnancy."]

Your best estimate will do.

[RANGE S12 +1 - 999]
Kilograms: |_|_|_|

99. [ ] I currently weigh the most I've weighed in the past 3 years [EXCLUSIVE]

[IF CURRENTLY MAX WEIGHT (S16/99) AUTO FILL S16 WITH S12]

BMI 30+ (S13 ≥ 30)
S17. In the past 6 months have you had significant weight loss due to major injury or illness (e.g., cancer, accident)?

1. Yes [TERMINATE]
2. No [CONTINUE]

BMI 30+ (S13 ≥ 30)
S18. HIDDEN QUESTION FOR PERCENT WEIGHT LOSS

PERCENT WEIGHT LOSS = ((S16 - S12) / S16)*100

[IF AT LEAST 5% WEIGHT LOSS (S18≥5) ASK S19. ELSE JUMP TO S22]

AT LEAST 5% WEIGHT LOSS (S18≥5)
S19. You indicated your current weight is less than your maximum weight within the past 3 years. For how long would you say you've been able to maintain your weight loss?

1. 6 months or less
2. More than 6 months but less than a year
3. A year or more
4. I have not been able to maintain weight loss

AT LEAST 5% WEIGHT LOSS (S18≥5)
S20. You indicated your current weight is less than your maximum weight within the past 3 years. How successful do you feel you have been in losing weight?

1. Not at all successful
2. Not very successful
3. Somewhat successful
4. Very successful
5. Extremely successful

BMI 30+ (S13 ≥ 30)
S21. HIDDEN QUESTION FOR WEIGHT LOSS STATUS
1. MAINTAINING WEIGHT LOSS (GET IF 10%+ LOSS AND MORE THAN 1 YEAR SUCCESS AND NOT ILLNESS/INJURY (S18 >= 10 AND S19/3 AND S17/2))
2. NOT CURRENTLY MAINTAINING WEIGHT LOSS (GET IF LESS THAN 10% LOSS OR NOT MORE THAN 1 YEAR SUCCESS (S18 < 10 OR S19/1,2,4 OR S17/1))

BMI 30+ (S13 ≥ 30)
S22. Which of the following statements best describes you today?
1. I am **not** concerned about my weight, and I **have no plans** for weight loss within the next 6 months
2. I am concerned about my weight, but I **have no plans** for weight loss within the next 6 months
3. I am aware I have excess weight, and am **seriously considering** taking action to lose weight
4. I am aware I have excess weight, and I intend to take action to lose weight **within the next month**
5. I am committed to/enrolled in a plan to lose weight
6. I have lost weight in the past year and have been able to keep it off
7. I have lost weight in the past year, but have **not** been able to keep it off

BMI 30+ (S13 ≥ 30)
S23. Have you spoken to your healthcare provider about a weight loss plan within the past 6 months?

1. Yes
2. No

HAS SPOKEN TO HCP ABOUT WEIGHT LOSS PLAN IN PAST 6 MONTHS (S23/1)
S24. Which of the following healthcare providers have you talked to about weight loss in the past 6 months?

Please select all that apply.
[RANDOMIZE]
1. My primary care physician
2. Dietitian
3. Obesity specialist
4. Psychologist or Psychiatrist
5. Other healthcare professional (a nurse, a physician who specializes in a certain condition, such as diabetes) [ANCHOR]

FINAL QUOTA QUESTIONS
S100 **TOTAL N =1,000**

1. PERSON WITH OBESITY N = 1,000
    a. AGE 18+ (S3>17)
    b. LIVES IN SWITZERLAND (S5/NE99)
    c. NOT PREGNANT (S4/1 or (S4/2-3 AND S9/2))
    d. DOES NOT PARTICIPATE IN INTENSE FITNESS OR BODY BUILDING PROGRAMS (S10/2)
    e. CURRENT BMI 30+ (S13 ≥ 30)
    f. DID NOT HAVE SIGNIFICANT WEIGHT LOSS OR GAIN IN PAST (S17/2)
    g. PROVIDED INCOME (S6/NE DECLINE)

2. NOT QUALIFIED N=9999

S101 REGION/LANGUAGE

1. GERMAN (S5A/1) N=TBD
2. FRENCH (S5A/2) N=TBD
3. ITALIAN (S5A/3) N=TBD

SOFT QUOTAS QUESTION
S102 COMMITTED TO ACTION

1. COMMITTED TO ACTION N = 9999
    a. INTEND TO TAKE ACTION TO LOSE WEIGHT OR COMMITTED TO/ENROLLED IN A PLAN TO LOSE WEIGHT (S22/4-5)

2. NOT COMMITTED TO ACTION N = 9999
    a. NOT ACTIVELY COMMITTED OR TAKING ACTION TO LOSE WEIGHT (S22/NE 4-5)

SOFT QUOTAS QUESTION
S120 – MAINTAINING WEIGHT LOSS SUCCESS

1. MAINTAINING WEIGHT LOSS (S21/1) N=9999
2. NOT CURRENTLY MAINTAINING WEIGHT LOSS (S21/2)N=9999

SOFT QUOTAS QUESTION
S125 WEIGHT CLASSIFICATION

1. Obesity Class I (S13 ≥ 30 AND S13 < 35) N=9999
2. Obesity Class II (S13 ≥ 35 AND S13 < 40) N=9999
3. Obesity Class III (S13 ≥ 40) N=9999

SECTION 100: PATIENT WEIGHT HISTORY / DEMOGRAPHICS

ALL RESPONDENTS (S100/1)
Q100. Thank you so much for your willingness to participate in this important research study – your input is very valuable! Please be aware that we may be asking some questions about your personal health.

We appreciate your open and honest feedback and want to assure you that your responses will be kept strictly confidential and only reported in summary with other respondents' data.

Which of the following best describes your current employment status?

1. Employed full-time
2. Employed part-time
3. Self-employed
4. Not employed, but looking for work
5. Not employed and not looking for work
6. Retired
7. Student
8. Permanent disability
9. Other
10. Decline to answer

ALL RESPONDENTS (S100/1)
Q102A. In general, would you say your health is:
[DO NOT SHOW #S ON SCREEN]

Excellent	Very Good	Good	Fair	Poor
1	2	3	4	5

ALL RESPONDENTS (S100/1)
Q103. Which of the following do you believe best describes your current weight?

1. Underweight
2. Normal weight
3. Overweight
4. Obesity
5. Extreme obesity

ALL RESPONDENTS (S100/1)
Q120. Have you discussed your weight and/or talked about losing weight with a healthcare provider (physician, nurse, etc.) in the past 5 years?

1. Yes
2. No

ALL RESPONDENTS (S100/1)
Q121A. [IF DISCUSSED (Q120/1) "Which of the following healthcare providers have you ever discussed your weight with?

Please consider all weight related conversations, regardless of who initiated the conversation."]

IF HASN'T DISCUSSED (Q120/2) "Which of the following healthcare providers would you consider discussing your weight with?"]

Please select all that apply.
[RANDOMIZE]

1. My primary care physician
2. Dietitian
3. Obesity specialist
4. Psychologist or Psychiatrist
5. Other healthcare professional (a nurse, a physician who specializes in a certain condition, such as diabetes) [ANCHOR]
6. [IF Q120/2 DISPLAY:I would not talk to a healthcare provider about my weight]

ALL RESPONDENTS (S100/1)
Q122A. Approximately how old were you when you first remember struggling with excess weight or obesity?

Your best estimate will do.

[RANGE 0-S3]
Age |_|_|

[IF DISCUSSED (Q120/1) ASK Q122. ELSE JUMP TO Q200]

HAS DISCUSSED WITH HCP (Q120/1)
Q122. Approximately how old were you when a healthcare provider first discussed your excess weight or recommended that you lose weight?

Your best estimate will do.

[RANGE Q122A-S3]
Age |_|_|

SECTION 200: READINESS TO CHANGE / PREVIOUS SUCCESS

ALL RESPONDENTS (S100/1)
Q200. Now, we would like to understand your overall goals concerning your weight and health. Please select the top 3 most important goals for you to personally achieve as part of your weight management, if any.

Please select only 3 items.

[SELECT 3] [RANDOMIZE]

1. Maintain current weight without gaining more
2. To lose (any amount of) weight
3. To lose a pre-specified % of my body weight
4. To lose a pre-specified number on the scale
5. To decrease the number of medications I must take
6. To improve my existing health condition(s)
7. To reduce the risks associated with excess weight / prevent a health condition
8. To have more energy
9. To improve my appearance
10. To buy / wear clothing that I like
11. To feel more confident / less judged by other people
12. To improve my sex life
13. To improve chances for career progression
14. To stay active with my family
15. To live a longer life
16. Other [ANCHOR]
17. None, I do not have any weight management goals [EXCLUSIVE] [ANCHOR]

HCP: Q225
ALL RESPONDENTS (S100/1)
Q203. Which of the following, if any, have motivated you the most to lose weight?

Please select all that apply.
[MULTIPLE RESPONSE] [RANDOMIZE WITHIN GROUP. RANDOMIZE GROUP ORDER, DON'T SHOW HEADINGS]

Physical Health
1. Having general health concerns
2. Wanting to stop or not need to take medication for a weight-related health condition
3. Reaching the upper end of the weight range I am comfortable with
4. A specific personal medical event (heart attack, stroke, etc.) or diagnosis (diabetes, liver disease, sleep apnea, etc.)

Support
5. Encouragement, support, recommendations from family or friends
6. Encouragement, support, recommendations from a healthcare provider
7. Encouragement, support, recommendations from wellness/fitness programs or a personal trainer
8. Encouragement, support from others who are trying to lose weight (losing weight with a spouse, work, health or fitness competition, etc.)

Appearance

9. Wanting to fit into a smaller clothing size
10. Wanting to be more fit/in better shape

Goals

11. Wanting to feel better physically, have more energy or be more active
12. Wanting to be more confident/improve my self-esteem
13. Wanting to improve my job performance
14. Wanting to improve my sex life
15. Wanting to be a positive role model for my family/children

Life Events

16. A major life change such as retirement, divorce, break-up, starting a family
17. An upcoming special occasion or event
18. A specific medical event (heart attack, stroke, etc.) or diagnosis (diabetes, liver disease, sleep apnea, etc.) in a family member/close friend

19. Other [ANCHOR]
20. None of the above/I have no desire to lose weight [EXCLUSIVE] [ANCHOR]

HCP: Q215

ALL RESPONDENTS (S100/1)

Q205. How many times in your adult life (after age 18) have you made a serious weight loss effort (e.g., followed a program, set goals, put your mind to it, or worked with a qualified healthcare professional), whether or not you were successful?

Please enter 0 if you have never made a serious weight loss effort. Please provide your best estimate.

[RANGE 0-99]
weight loss effort(s) |_|_|_|

[IF MADE WEIGHT LOSS EFFORT (Q205/ >0) ASK Q210A. ELSE JUMP TO Q245b.]

HCP: Q515

MADE WEIGHT LOSS EFFORT (Q205/ >0)

Q210A. Which of the following methods for managing your weight have you ever **discussed** with a healthcare provider?

Please select all that apply.

[MULTI-SELECT, RANDOMIZE ROWS WITHIN GROUP, RANDOMIZE GROUP ORDER EXCEPT 16 AND 99 ITEMS, DON'T SHOW HEADINGS]

Diet / Healthy Eating

1. General improvement in eating habits / reducing calories
2. Specific diet or diet program (for example InShape, Weight Watchers, Metabolic Balance, Pronokal)
3. Elimination diets (avoiding fats, sugary beverages, carbohydrates, etc.)

Exercise

4. Generally, be more active / increase physical activity
5. A formal exercise program / gym membership / personal trainer

Tracking
6. Meal / nutrient tracking (on paper or an app)
7. Exercise tracking (on paper or app such as smartphone apps, wearable fitness tracker, etc.)

Medical Treatment / Medication
8. Over-the-counter (non-prescription) weight loss medication (vitamins, supplements, etc.)
9. Prescription weight loss medication
10. Visiting a dietitian (non-physician)
11. Visiting an obesity specialist
12. Behavior therapy or psychotherapy such as counseling or behavior modification
13. Weight loss surgery / bariatric surgery

Quality of life management
14. Stress management
15. Sleep quality management

16. Other [ANCHOR]
99. None of the above [EXCLUSIVE][ANCHOR]

DISCUSSED ANY METHOD OF MANAGING WEIGHT WITH AN HCP (Q210A/NE 99)
Q211. For each of these methods for managing your weight you have **ever discussed** with a healthcare provider, which type of healthcare provider did you have the discussion with?

[DISPLAY AS GRID WITH HCP TYPES IN COLUMNS, CODES SELECTED AT Q210A AS ROWS]
[PN: ALLOW FOR MULTIPLE SELECTIONS PER ROW; DO NOT RECALL IS EXCLUSIVE IN ROW]
[DISPLAY ROWS IN SAME ORDER AS Q210A, DON'T SHOW HEADINGS]
[COLUMN HEADERS: PCP/GP Obesity specialist Other Specialist Dietitian Do Not Recall

Diet / Healthy Eating
1. General improvement in eating habits / reducing calories
2. Specific diet or diet program (for example InShape, Weight Watchers, Metabolic Balance, Pronokal)
3. Elimination diets (avoiding fats, sugary beverages, carbohydrates, etc.)

Exercise
4. Generally, be more active / increase physical activity
5. A formal exercise program / gym membership / personal trainer

Tracking
6. Meal / nutrient tracking (on paper or an app)
7. Exercise tracking (on paper or app such as smartphone apps, wearable fitness tracker, etc.)

Medical Treatment / Medication
8. Over-the-counter (non-prescription) weight loss medication (vitamins, supplements, etc.)
9. Prescription weight loss medication

10. Visiting a dietitian (non-physician)
11. Visiting an obesity specialist
12. Behavior therapy or psychotherapy such as counseling or behavior modification
13. Weight loss surgery / bariatric surgery

Quality of life management
14. Stress management
15. Sleep quality management
16. Other [ANCHOR]
99. None of the above [EXCLUSIVE][ANCHOR]

MADE WEIGHT LOSS EFFORT (Q205/ >0)
Q210C. Which of the following methods for managing your weight **are you currently trying**?

Please select all that apply.

[INSERT FULL LIST FROM Q210A. SHOW IN SAME ORDER.]

99. [ ] I am not currently trying any of these methods [EXCLUSIVE]

MADE WEIGHT LOSS EFFORT (Q205/ >0)
Q210D. Which of the following methods for managing your weight do you think are **effective for weight loss**?

Please select all that apply.

[INSERT LIST FROM Q210A. SHOW IN SAME ORDER.]

Diet / Healthy Eating
1. General improvement in eating habits / reducing calories
2. Specific diet or diet program (for example InShape, Weight Watchers, Metabolic Balance, Pronokal)
3. Elimination diets (avoiding fats, sugary beverages, carbohydrates, etc.)

Exercise
4. Generally, be more active / increase physical activity
5. A formal exercise program / gym membership / personal trainer

Tracking
6. Meal / nutrient tracking (on paper or an app)
7. Exercise tracking (on paper or app such as smartphone apps, wearable fitness tracker, etc.)

Medical Treatment / Medication
8. Over-the-counter (non-prescription) weight loss medication (vitamins, supplements, etc.)
9. Prescription weight loss medication
10. Visiting a dietitian (non-physician)
11. Visiting an obesity specialist
12. Behavior therapy or psychotherapy such as counseling or behavior modification

13. Weight loss surgery / bariatric surgery

Quality of life management
14. Stress management
15. Sleep quality management

16. Other [ANCHOR]
99None of the above [EXCLUSIVE][ANCHOR]

MADE WEIGHT LOSS EFFORT (Q205/ >0)
Q213. Have you ever had what you would consider a successful weight loss effort, but later regained the weight after keeping it off for at least 6 months?

1. Yes
2. No

ALL RESPONDENTS (S100/1)
Q245B. As an outcome of a weight loss effort, what weight would you set for yourself as a goal?

[RANGE 1-999]
Kilograms: |_|_|_|

ALL RESPONDENTS (S100/1)
Q245C HIDDEN CALCULATION FOR % WEIGHT LOSS

[Q245C = (100 * S12 – Q245B) / S12]

ALL RESPONDENTS (S100/1)
Q246. How much do you agree with the following statements?

Strongly disagree 1	Disagree 2	Neutral 3	Agree 4	Strongly agree 5

[RANDOMIZE LIST AND GROUPS, DON'T SHOW HEADINGS]

COPD Helplessness Index - ADAPTED
1. No matter what I do or how hard I try, I just cannot seem to overcome my weight issues
2. When it comes to managing my weight, I feel I can only do what my physician tells me to do
3. My weight is controlling my life
4. It seems as though fate and other factors beyond my control affect my weight

Dietary Helplessness and Disinhibition in Weight
5. No matter how hard I try to change, I end up falling back into some of my old eating habits

SECTION 400: OBESITY AWARENESS AND PERCEPTIONS

HCP: Q405
ALL RESPONDENTS (S100/1)
Q405. In general, how large of an impact do you believe the following health conditions have on a person's overall health?

Use a scale where 1 means "Very little impact" and 5 means "An extreme impact."

1 - Very little impact 2 3 4 5 - An extreme impact99 - Not familiar with this condition

[RANDOMIZE]
1. Diabetes
2. Chronic obstructive pulmonary disease (COPD)
3. Cancer
4. Stroke
5. Obesity (BMI of 30 or greater)

ALL RESPONDENTS
Q405A HIDDEN QUESTION
[RECODE Q405 ATTRIBUTES 1-4 BASED ON IF THEY'RE GREATER OR LESS THAN OBESITY (Q405_5)]

OBESITY IS MORE SERIOUS (Q405_ATTRIBUTE < Q405_5)
OBESITY IS AS SERIOUS (Q405_ATTRIBUTE = Q405_5)
OBESITY IS LESS SERIOUS (Q405_ATTRIBUTE > Q405_5)

ALL RESPONDENTS (S100/1)
Q415. Assuming you remain at your current weight, how much do you worry that your weight may affect your health in the future?

1. Not at all
2. A little
3. Somewhat
4. A lot
5. An extreme amount

ALL RESPONDENTS (S100/1)
Q420 Compared to a person who is not overweight, how easy or difficult do you think each of the following is for someone who is overweight?

[RANDOMIZE]	Much harder	Somewhat harder	About the same	Somewhat easier	Much easier

Getting a job	1	2	3	4	5
Advancing/promotion in a job	1	2	3	4	5
Making friends	1	2	3	4	5
Forming romantic relationships	1	2	3	4	5

ALL RESPONDENTS (S100/1)
Q425 How much of an impact do you think a person being overweight has on each of the following?

[RANDOMIZE]	Very negativ e impact	Somewh at negative impact	No impac t	Somewh at positive impact	Very positive impact
How smart people think the person is	1	2	3	4	5
How athletic people think the person is	1	2	3	4	5
How healthy people think the person is	1	2	3	4	5
Relationships with friends	1	2	3	4	5
Relationships at home/with family	1	2	3	4	5
How much ambition people think the person has	1	2	3	4	5
How much willpower people think the person has	1	2	3	4	5

ALL RESPONDENTS (S100/1)
Q435. For quality control purposes, please select No.

1. Yes
2. No
3. Maybe

SECTION 500: OBESITY ATTITUDINAL QUESTIONS

HCP: Q503
ALL RESPONDENTS (S100/1)
Q500. Please indicate how much you agree with each of the following…

Use a scale where 1 means "Do not agree at all" and 5 means "Completely agree".

1 - Do not agree at all 2 3 4 5 - Completely agree

[RANDOMIZE, CAROUSEL]
1. It is easy for me to lose weight.
2. I could lose weight if I really set my mind to it.
3. If I lost weight, it would be easy for me to keep the weight off.
4. I know how to lose weight.
5. My healthcare provider has a responsibility to actively contribute to a successful weight loss effort.
6. My weight loss is completely my responsibility.
7. For me to lose weight, I would need to completely change my lifestyle.
8. I am happy with my current weight.
9. I am past the point where I can lose weight on my own.
10. I am motivated to lose weight.
11. For quality control purposes, please select 1.
12. Obesity is less important to me than other diseases.
13. I do not feel comfortable bringing up my weight unless my healthcare provider mentions it first.
14. There is nothing my doctor can do to help me manage my weight.
15. I know how to keep the weight off. [SHOW AFTER CODE 4]

HCP: Q507
ALL RESPONDENTS (S100/1)
Q507. How much do you agree that each of the following is a barrier to you losing weight?

Please use a scale where 1 means "Do not agree at all" and 5 means "Completely agree".

1 - Do not agree at all 2 3 4 5 - Completely agree

[RANDOMIZE, CAROUSEL]

1. My preference for unhealthy food
2. Lack of exercise
3. My genes (e.g., inherited from my family)
4. The nature of my job / employment [SHOW IF EMPLOYED (Q100/1-3)]
5. A lack of time to cook healthy meals
6. My other health conditions
7. My friends and family
8. My healthcare provider
9. My finances
10. My lack of motivation
11. My lack of ability to control my hunger

12. The cost of healthy food
13. Limited access to healthy food
14. My mental health / emotional status
15. Fear of failure
16. Limited coverage for health care costs
17. Limited mobility due to physical health problems
18. The possibility of regaining the weight
19. My unhealthy eating habits (large portion sizes, excessive snacking)
20. My lack of understanding of what obesity is
21. The cost of weight management medications, programs and services
22. My metabolism
23. My age

HCP: Q521
ALL RESPONDENTS (S100/1)
Q520. Please indicate how much you agree with the following regarding prescription medications for weight loss…

Use a scale where 1 means "Do not agree at all" and 5 means "Completely agree".
[CAROUSEL]

1 - Do not agree at all 2 3 4 5 - Completely agree 99 Don't know/ Not sure

1. A weight loss medication available by prescription from my physician would be more effective than other treatment options for weight loss.
2. If I heard of a new prescription weight loss medication, I would ask my physician to prescribe it to me.
3. I would rather take a prescription medication than have a surgery (bariatric) to lose weight.
4. I am concerned about the side effects associated with prescription weight loss medications.
5. I would like my healthcare provider to offer me a prescription weight loss medication to help me with my weight loss efforts.
6. I am familiar with prescription weight loss medications.
7. Cost is a major barrier for me to consider using prescription weight loss medications.
8. I would rather lose weight myself than depend on medication.

HCP: Q525
ALL RESPONDENTS (S100/1)
Q525. Please indicate how much you agree with the following regarding weight loss surgery…

Note that weight loss surgery is also known as bariatric surgery.
[CAROUSEL]

Use a scale where 1 means "Do not agree at all" and 5 means "Completely agree".

1 - Do not agree at all 2 3 4 5 - Completely agree 99 Don't know/ Not sure

1. Weight loss surgery is more effective than other treatment options for weight loss.

2. I would rather have weight loss surgery than change my lifestyle to lose weight.
3. I have concerns about the safety of having weight loss surgery.
4. Cost is a major barrier for me when considering weight loss surgery.
5. The wait time for weight loss surgery is too long.
6. I would rather lose weight with diet and exercise than have weight loss surgery.
7. Having weight loss surgery is the “easy” way out.
8. After weight loss surgery, the weight may come back.

SECTION 600: SUPPORT STRUCTURE

HCP: Q650

ALL RESPONDENTS (S100/1)

Q640. Please indicate how much you agree with the following statements regarding obesity and weight management.

Use a scale where 1 means “Do not agree at all” and 5 means “Completely agree”.

1 - Do not agree at all 2 3 4 5 - Completely agree 99 - Does not apply

[RANDOMIZE]

1. Maintaining a healthy weight is a priority for our country’s healthcare system.
2. Cost of obesity therapy / treatment is a barrier to me losing weight.
3. I feel the healthcare system (doctor’s offices, hospitals, etc.) is a good resource for those looking to lose weight.
4. [DISPLAY IF CURRENTLY EMPLOYED BUT NOT SELF-EMPLOYED (Q100/1-2)] My employer is an important partner in my efforts to manage my weight.
5. Obesity is a chronic disease.
6. A loss of 5-10% body weight would be extremely beneficial to my overall health.
7. The treatment of obesity should be a team effort between different healthcare professionals.
8. For quality control purposes, please select 3.

SECTION 700: INTERACTION WITH HCP

[IF HAS DISCUSSED EXCESS/LOSING WEIGHT WITH AN HCP (Q120/1) ASK Q700. ELSE JUMP TO Q702.]

HAS DISCUSSED EXCESS/LOSING WEIGHT WITH HCP (Q120/1)

Q700. Have you ever been diagnosed with obesity by a medical doctor or qualified healthcare professional?

1. Yes
2. No

HCP: Q702

HAS DISCUSSED EXCESS/LOSING WEIGHT WITH HCP (Q120/1)
Q701. Who typically brings up your weight during your appointments?

1. I usually start the conversation.
2. My healthcare provider usually starts the conversation.

HCP: Q702
ALL RESPONDENTS (S100/1)
Q702. [IF HCP HAS BROUGHT UP WEIGHT (Q701/2) "Do you like that your healthcare provider brings up your weight during appointments?"]

[IF HCP HAS NOT BROUGHT UP WEIGHT (Q701/NE2 OR Q120/2) "Would you like for your healthcare provider to bring up your weight during appointments?"]

1. Yes
2. No

HCP: Q725
HAS DISCUSSED WEIGHT WITH HCP (Q120/1)
Q710. Thinking about your most recent discussion, how did you feel after discussing your weight with your healthcare provider?

Please select all that apply.

[MULTI-SELECT] [RANDOMIZE]
1. Motivated
2. Hopeful
3. Supported
4. Embarrassed
5. Discouraged
6. Blamed
7. Offended
8. Confused
9. Relieved
10. Indifferent
11. Rushed
12. Other [ANCHOR]

HCP: Q720
ALL RESPONDENTS (S100/1)
Q720. [IF DISCUSSED (Q120/1) "What types of weight management goals have you set with your healthcare provider?"]

IF HASN'T DISCUSSED (Q120/2) "What types of weight management goals would you like to set with your healthcare provider?"]

Please select all that apply.

[RANDOMIZE]

1. To not gain any more weight

2. To lose weight (did not specify an amount)
3. To lose a pre-specified % of my body weight
4. To lose a pre-specified number on the scale
5. To decrease the number of medications I must take
6. To improve my existing health condition(s)
7. To reduce the risks associated with weight / prevent a health condition
8. To have more energy
9. To improve my appearance
10. Short-term (within the next 6 months) weight loss goals
11. Long-term (more than six months from now) weight loss goals
12. To improve my lifestyle
13. To reduce my stress and improve overall health and well-being
14. To improve my physical and mental health and well-being
15. Other [ANCHOR]
16. [IF DISCUSSED (Q120/1) "I have not set any goals with my healthcare provider" IF HASN'T DISCUSSED (Q120/2) "I would not like to set any goals with my healthcare provider"] [EXCLUSIVE][ANCHOR]

IF DISCUSSED AND HCP RECOMMENDED LOSS OF PERCENT BODY WEIGHT (Q120/1 AND Q720/3) ASK Q725. ELSE JUMP TO PN BEFORE Q726]

DISCUSSED AND HCP RECOMMENDED LOSS OF PERCENT BODY WEIGHT (Q120/1 AND Q720/3)
Q725. You mentioned your healthcare provider suggested you lose a percentage of your body weight. What percent did they suggest you try to lose?

[Range 1-100]
Percent of body weight |_|_|_|%

[IF DISCUSSED AND HCP RECOMMENDED LOSING WEIGHT (Q120/1 AND Q720/4) ASK Q726. ELSE JUMP TO Q742]

DISCUSSED AND HCP RECOMMENDED TARGET WEIGHT LOSS (Q120/1 AND Q720/4)
Q726. You mentioned your healthcare provider suggested you lose a certain number of kilograms. How many kilograms did they suggest you try to lose?

[RANGE 1-999]
Kilograms: |_|_|_|

ALL RESPONDENTS (S100/1)
Q742. Please indicate how much you agree with the following statements:

Use a scale where 1 means "Do not agree at all" and 5 means "Completely agree".

1 - Do not agree at all 2 3 4 5 - Completely agree

[RANDOMIZE] [SHOW 1-6 IF DISCUSSED (Q120/1]
1. I feel comfortable talking to my healthcare provider about my weight.

2. My healthcare provider listens carefully to what I have to say about my weight.
3. My healthcare provider understands the difficulties of weight management.
4. I trust my healthcare provider's advice when it comes to weight management.
5. I follow my healthcare provider's advice about weight management.
6. It is important to me that my healthcare provider is at a healthy weight.

[RANDOMIZE] [SHOW 7-12 IF HASN'T DISCUSSED (Q120/2]

7. I would feel comfortable talking to my healthcare provider about my weight.
8. I expect my healthcare provider would listen carefully to what I have to say about my weight
9. I expect my healthcare provider would understand the difficulties of weight management.
10. I would trust my healthcare provider's advice when it comes to weight management.
11. I would follow my healthcare provider's advice about weight management.
12. It is important to me that my healthcare provider is at a healthy weight.

HCP: Q740
ALL RESPONDENTS (S100/1)
Q759. [IF DISCUSSED (Q120/1) "Did your healthcare provider schedule a follow-up appointment or call related to your weight after your last visit?"]

[IF HASN'T DISCUSSED (Q120/2) "Would you like your healthcare provider to schedule a follow-up appointment or call related to your weight after you visit?"]

1. Yes
2. No

IF DISCUSSED AND SCHEDULED FOLLOW UP (Q120/1 AND Q759/1) ASK Q760]

HCP: Q740
DISCUSSED AND SCHEDULED FOLLOW UP (Q120/1 AND Q759/1)
Q760. Did you attend the follow-up appointment with your healthcare provider related to your weight, as scheduled?

1. Yes
2. No, and I don't intend to
3. No, but I am planning to

HCP: Q708
ALL RESPONDENTS (S100/1)
Q770. Which of the following are/would be the **top five** reasons for which you **might not** discuss managing your weight with your healthcare provider?

Please select up to 5 items only.

[MULTISELECT 5 ANSWERS, RANDOMIZE]

1. The appointment is not long enough / I'm rushed
2. There are more important health issues / concerns to discuss
3. I do not feel comfortable bringing it up
4. I do not trust and/or do not have a close relationship with my healthcare provider
5. I do not see my weight as a significant medical issue

6. I am in good health and do not have weight-related health problems
7. I believe it is my responsibility to manage my weight
8. I am not interested in losing weight
9. I do not feel motivated to lose weight
10. I do not believe I am able to lose weight
11. Even if I were to lose weight, I would just gain it back
12. I already know what I need to do to manage my weight
13. There is nothing my healthcare provider can do to help me manage my weight
14. I do not think my healthcare provider is interested in / concerned about my weight
15. I have had previous bad experience discussing weight with a healthcare provider
16. I do not have the financial means to support a weight loss effort
17. My healthcare provider does not have training to provide weight management services
18. My healthcare provider's office is not set up to treat patients with excess weight / obesity
19. Other [ANCHOR]

SECTION 800: SOLUTION REVIEW

HCP: Q760
ALL RESPONDENTS (S100/1)
Q825. Please select top 3 types of information that would be most helpful for you personally in managing your weight.

Please select only three items.
Information on…

[SELECT 3 ONLY]
[RANDOMIZE]
1. Healthy ways to lose weight
2. The health benefits of weight loss
3. Medical treatment options for weight management
4. Managing weight with exercise
5. How to maintain weight loss
6. How healthcare providers can help with weight management
7. Stress management techniques
8. Realistic weight loss goals
9. Exercises which are safe to do for people with mobility limitations
10. Healthy vs. non-healthy eating

HCP: Q602
ALL RESPONDENTS (S100/1)
Q826. What are the **top 5 types of support that would be most helpful for you personally** to be successful with managing your weight?

Select your top 5.

[5 SELECTIONS] [RANDOMIZE]
1. Resources for family and friends to help understand how to be supportive
2. Specific meal plans to follow for weight management

3. Online support groups for those trying to lose weight
4. Local in-person support groups for those trying to lose weight
5. Motivational programs to help people stay on track with weight loss plan
6. More programs offered at work to help people lose weight
7. Encouragement from friends/family to increase desire to keep going
8. Financial support for healthy choices (gym membership, healthy foods)
9. Diary for tracking weight over time (paper based or electronic)
10. Diary for tracking food intake (paper based or electronic)
11. Diary for tracking physical activity (paper based or electronic)
12. App with weight loss tracking and ideas for healthy eating and physical activity
13. Programs for physical activity
14. Prescription drugs for weight loss
15. Over-the-counter drugs for weight loss
16. Personal trainer / weight loss counselor
17. Weekly follow-up with a healthcare provider
18. Meetings with dietitian (non-physician)
19. A work culture that encourages a healthy lifestyle
20. Access to mental health support
21. Access to stress management support
22. Access to a physician who specializes in obesity
23. Other [ANCHOR]
24. I don't need any of these types of support [EXCLUSIVE, ANCHOR]
25. Bariatric surgery

SECTION 900: DEMOGRAPHICS

HCP: Q906

ALL RESPONDENTS (S100/1)

Q900. Thank you again for your time so far. As we noted at the beginning of this survey, your personal information will never be shared with other organizations. Your honest answers are very much appreciated. Your responses to this survey will help the sponsor, Novo Nordisk, understand the healthcare experiences of patients and healthcare providers.

To finish, we would like to gather some additional information used for categorization purposes.

Which of the following medical conditions have you ever been diagnosed with by a healthcare provider?

Please select all that apply.

[ALPHA SORT]

1. Cardiovascular Diseases: Coronary artery disease/coronary heart diseases/congestive heart failure, pulmonary embolism, stroke
2. Depression/Anxiety
3. High cholesterol (Dyslipidemia / triglycerides)
4. High blood pressure (Hypertension)
5. Infertility
6. Liver disease (e.g., Non-alcoholic fatty liver disease)
7. Obstructive Sleep Apnea
8. Osteoarthritis

9. Metabolic syndrome
10. Stomach or intestinal problems
11. Pre-diabetes
12. Diabetes (Type II)
13. Cancer
14. Polycystic Ovary Syndrome (PCOS) [ONLY SHOW IF FEMALE OR OTHER (S4/2-3)]
15. Eating disorder (e.g. binge eating disorder, night eating syndrome)
16. Other condition [ANCHOR]
17. None of these [EXCLUSIVE] [ANCHOR]

ALL RESPONDENTS (S100/1)
Q930. Have you ever had bariatric surgery?

1. Yes
2. No

EVER HAD BARIATRIC SURGERY (Q930/1)
Q932. How many years ago did you have bariatric surgery?

If you have had bariatric surgery less than one year ago, please enter "1" (one)

[RANGE 1-S3]
|_|_|_| # of years ago had bariatric surgery

ALL RESPONDENTS (S100/1)
Q901. How frequently do you weigh yourself?

Please select the answer that best applies.

1. Every day
2. 2-3 times a week
3. Once a week
4. Once every two weeks
5. Once a month
6. Once every two months
7. A few times a year
8. Never

ALL RESPONDENTS (S100/1)
Q902. In a typical week, how frequently do you exercise for at least a 20-minute period?

1. Never
2. Less than once a week
3. 1 to 2 times a week
4. 3 to 4 times a week
5. 5 to 6 times a week
6. 7 or more times a week

HCP: Q920

ALL RESPONDENTS (S100/1)
Q929. Which of the following best describes the size of the area in which you live?

1. City (urban center of influence)
2. Urban area close to a city
3. Rural area / village

SURVEY QUESTIONS: HCPs

Highlighting color key:
Question asked in ACTION IO
Question asked in ACTION IO, modified for Switzerland
New Question, not asked in ACTION IO

KJT Group, Inc.
6 East St
Honeoye Falls, NY, 14472

Awareness, Care & Treatment In Obesity MaNagement - ACTION Switzerland - HCP Questionnaire

KJT Group Contacts:

[Name redacted]
Email: [redacted]
Phone: [redacted]

[Name redacted]
Email: [redacted]
Phone: [redacted]

[Name redacted]
Email: [redacted]
Phone: [redacted]

[Name redacted]
Email: [redacted]
Phone: [redacted]

[Name redacted]
Email: [redacted]
Phone: [redacted]

Recruitment:

Respondent Group	Switzerland
PCPs	n=75
Non-PCP Specialties	n=50
Dietitians	n=25
**Total HCPs**	**n=150**

Screening Criteria:

All HCPs:

- Physician
- Specialty is **NOT** plastic surgeon, general surgeon
- Spends at least 50% of time in patient medical management (as opposed to surgical procedures, office procedures or research/administrative tasks)
- Practices in Switzerland
- In practice 2+ years
- Has seen at least 100 patients in past month (for all physicians EXCEPT psychiatrists)

- Has seen at least 10 unique patients in past month who have obesity (for psychiatrists has seen at least 5 unique patients in past month who have obesity): defined as a patient with a Body Mass Index (BMI) ≥30 with or without comorbidities. Physician only has to have *seen* this minimum # of patients, does NOT need to be treating them for obesity.

Dietitians
- Is an active member of the SVDE ASDD (Swiss Association of Dietitians)
- Practices in Switzerland
- In practice at least 2 years
- Seen at least 5 unique patients in past month who have obesity (BMI of 30 or greater)
- Spends at least 50% of time in patient care
- Counsels/treats patients with obesity (BMI of 30 or greater)
- Their services are covered by the mandatory basic health insurance (OKP)

Specialty Quota (Hard Quota):
- **Primary Care Providers**
  - Specialty is Family Practice, General Practice, General Internal Medicine (GIM who is focused on primary care)
- **Non-PCP Specialties**
  - Specialty is Obstetrics/Gynecologists, Endocrinologist/Diabetologists, Psychiatrists or General Internal Medicine (GIM who is focused on treatment of Diabetes and/or Obesity)

Obesity Specialist Definition (will fall out naturally):
- 50% or more of patients are seen for obesity/weight management (S12)

OR
- works in an obesity center/clinic (S15/6)

Targeting based on practice setting, years in practice, region, and title

Data Validation Questions:

Q240, Q503_11, Q650_8

SECTION S: SCREENER

ALL RESPONDENTS
S0 Thank you for taking the time to participate in this study. For your convenience we are offering this survey in multiple languages. Please select a language that you are most familiar with.

5. English (United Kingdom)
6. French
7. German
8. Italian

ALL RESPONDENTS
S1. Thank you for your interest in this survey. We appreciate your willingness to participate in this study on healthcare issues. For better legibility we abstain from consistently writing all possible gender forms throughout the survey. However, please know that all genders are equally considered.

Before participating, KJT Group requires you to review the following information:

- KJT Group is a **global market research company** requesting your consent to participate in this research survey. KJT Group is the data controller
- KJT's legal basis for the processing of your personal data is your consent
- Your participation is fully voluntary; you can choose to stop at any time and you can, after completion of the questionnaire, withdraw your consent at any time by writing to [KJT email redacted] without any penalty or loss of benefits to which you are otherwise entitled.
- The survey complies with all Swiss and European data protection regulations.
- The purpose of the survey is to help the sponsor (Novo Nordisk) understand the healthcare experiences of patients from the point of view of the HCPs.
- The risk of the research is to your privacy. Your responses will be kept **completely confidential** and will never be connected with your name. Your alternative is to not participate in the survey.
- You have the **right to see and get a copy of your data, amend your data, or erase your data** at any time.
- We expect, on average, it will take about **25 minutes** to complete this survey..
- Your responses will be aggregated with other responses in KJT Group's research report and will be made publicly available in a peer-reviewed scientific journal publication once the study has finished. However, **your name will never be included in the report, publication, or identified to the sponsor**.
- Your responses will be transferred to and **stored on secure servers in the United States**.
- We will **destroy your personally identifiable information within 12 months of data collection**; however, we will maintain a permanent record of this consent.
- If you do not qualify for the study, your personal data will be stored electronically by KJT Group and erased no later than 12 months after the end of data collection.

- All information from this study will be stored for at least 5 years after the Study Report is made, or according to local requirements. The study report contains the full results of the study.
- Your **participation is voluntary,** and you may choose to stop participating at any time (withdraw consent) without any penalty or loss of benefits to which you are otherwise entitled.
- If you qualify and complete this survey, you will receive online panel credit/the honoraria listed in your invitation. There are no costs to you for your participation.
- Your personal information is protected by the data protection law as applicable in your country.
- KJT will make sure that the information we ask about you cannot be looked at by people who are not authorized to do so.

You may contact [KJT email redacted] with questions or concerns, or if you would like to follow-up on these points or need additional support.

Do you consent to these terms and wish to continue with the survey?

3. Yes [CONTINUE]
4. No [TERMINATE]

ALL RESPONDENTS
S2 To begin, we would like to gather some basic information to be used for categorization purposes.

Are you…?

1 Male
2 Female
3 Other

ALL RESPONDENTS
S3 In what year were you born?

Please enter as a four-digit number, e.g., 1963.

[RANGE: 1890-2020]
|_|_|_|_|

ALL RESPONDENTS
S4 HIDDEN COMPUTE FOR AGE

[IF AGE 18 OR OLDER (S4>17) ASK S5. ELSE, TERMINATE]

OLDER THAN 18 (S4>17)
S5 In what canton is the place of work where you spend most of your time located?

31) Aargau
32) Appenzell Ausserrhoden

33) Appenzell Innerrhoden
34) Basel-Landschaft
35) Basel-Stadt
36) Bern - French speaking region
37) Bern - German speaking region
38) Fribourg - French speaking region
39) Fribourg - German speaking region
40) Genève
41) Glarus
42) Graubünden - German or Romansh speaking region
43) Graubünden - Italian speaking region
44) Jura
45) Luzern
46) Neuchâtel
47) Nidwalden
48) Obwalden
49) Schaffhausen
50) Schwyz
51) Solothurn
52) St. Gallen
53) Thurgau
54) Ticino
55) Uri
56) Valais - French speaking region
57) Valais - German speaking region
58) Vaud
59) Zug
60) Zurich

99. I do not work in Switzerland [ANCHOR] [TERMINATE]

[IF PRACTICES IN SWITZERLAND (S5/NE99) CONTINUE. ELSE TERMINATE]

HIDDEN VARIABLE:
S5A [REGIONAL RECODES]

4. GERMAN [S5/1-5,7,9,11,12,15,17-23,25,27,29,30]
5. FRENCH [S5/6,8,10,14,16,26,28]
6. ITALIAN [S5/13,24]

PRACTICES IN TARGET COUNTRY (S5/NE99)

S6 Which of the following best describes your title?

1. Physician — CONTINUE
2. Dietitian — CONTINUE
3. Nurse — TERMINATE
4. Pharmacist (Pharm.D.) — TERMINATE
5. Other [ANCHOR] — TERMINATE

[PHYSICIAN OR DIETITIAN (S6/1,2) CONTINUE]

DIETITIAN (S6/2)
S6B Are you a member of SVDE ASDD (Swiss Association of Dietitians)?

1. Yes [CONTINUE]
2. No [TERMINATE]

PHYSICIAN (S6/1)
S7 What best describes your primary medical specialty?

[ALPHA SORT]
9. Family Practice Physician [CONTINUE]
10. General Practice Physician [CONTINUE]
11. General Internal Medicine (with focus on primary care) [CONTINUE]
12. General Internal Medicine (with focus on diabetes and/or obesity) [CONTINUE]
13. General Internal Medicine (with focus *other than* primary care, diabetes, obesity) [TERMINATE]
14. Bariatric Surgery [CONTINUE]
15. General Surgery [TERMINATE]
16. Obstetrics and Gynecology [CONTINUE]
17. Endocrinology/Diabetology [CONTINUE]
18. Cardiology [TERMINATE]
19. Gastroenterology [TERMINATE]
20. Plastic Surgery [TERMINATE]
21. Psychiatry [CONTINUE]
22. Other [TERMINATE] [ANCHOR]

[QUALIFY IF: FP, GP, GIM – Primary Care, GIM – diabetes/obesity, ENDO/DIAB, OB/GYN, PYSCHIATRY (S7/1,2,3,4,8,9,13)]
[IF BARIATRIC SURGERY (S7/6) ASK S7B, ELSE SKIP TO S8]

BARIATRIC SURGERY (S7/6)
S7B Do you manage patients with obesity beyond bariatric surgery (in addition to pre- and post-operative care)?

1. Yes
2. No

[IF BARIATRIC SURGEON DOES **NOT** MANAGE PATIENTS WITH OBESITY (S7B/2), TERMINATE]
[IF BARIATRIC SURGEON **DOES** MANAGE PATIENTS WITH OBESITY (S7B/1), ASK S8]

PHYSICIAN (S6/1) IN QUALIFYING SPECIALTY (S7/1,2,3,4,8,9,13) OR (S7/6 AND S7B/1))
S8 What percentage of your professional time is spent performing each of the following activities?

Your best estimate will do. Your responses must sum to 100%.

[SHOW TOTAL SUM INDICATOR; MUST TOTAL TO 100]
[RANGE 0-100]
1. Patient care/medical management I_I_I_I%
2. Surgical procedures I_I_I_I%

3. Outpatient procedures (endoscopies, imaging, etc.) I_I_I_I%
4. Research or administrative tasks I_I_I_I%

[IF S7/1,2,3,4,8,9,13 AND AT LEAST 50% TIME IN PATIENT CARE/MEDICAL MANAGEMENT (S8_1 >49) CONTINUE; IF BARIATRIC SURGEON AND MANAGES PATIENTS WITH OBESITY (S/7/6 AND S7B/1) AND AT LEAST 50% OF TIME IN PATIENT CARE AND SURGICAL (S8/1 + S8/2 >49 CONTINUE, OTHERWISE TERMINATE]

DIETITIAN WHO IS MEMBER OF SVDE (S6/2 AND S6B/1)
S8B What percentage of your professional time is spent performing each of the following activities?

Your best estimate will do. Your responses must sum to 100%.

[SHOW TOTAL SUM INDICATOR; MUST TOTAL TO 100]
[RANGE 0-100]
1. Patient care/management I_I_I_I%
2. Research, reporting or administrative tasks I_I_I_I%

[IF S6/2 AND S6B/1 AND AT LEAST 50% TIME IN PATIENT CARE/MEDICAL MANAGEMENT (S8b_1 >49) CONTINUE]

SPENDS 50%+ OF TIME IN DIRECT PATIENT CARE (S8/1>49 OR S8b_1>49)
S9 How many years have you been in practice [PHYSICANS (S8/1>49):since the end of your post-graduate training (FMH specialist or practising physician)]?

[IF PHYSICIAN DISPLAY(S6/1): *If you are still in your post-graduate training (FMH specialist or practising physician) , or have not been in practice for at least one year, please enter "0" (zero).*]

[IF DIETITIAN (S6/2) DISPLAY: *If you have not been in practice for at least one year, please enter "0" (zero).*]

[RANGE: 0-50]
Years in practice |_|_|_|

[IF IN PRACTICE 2+ YEARS (S9/2+) GO TO S10, ELSE TERMINATE]

IN PRACTICE 2+ YEARS (S9/2+)
S10 In the past month, approximately how many total adults (age 18 and older) did you personally see/treat across all conditions and across all care settings (hospitals, outpatient clinics, practice, etc.)?

Your best estimate is fine.
[RANGE: 0-9999]
Patient(s) in past month [_|_I_I_]

[IF PHYSICIAN AND SEEN AT LEAST 100 PATIENTS (S6/1 AND S10>99) ASK S11. IF DIETICIAN AND SEEN AT LEAST 50 PATIENTS (S6/2 AND S10>49) ASK S11, ELSE TERMINATE]

PHYSICIAN IN QUALIFIED SPECIALTY BUT *NOT* PSYCHIATRIST AND SEEN AT LEAST 100 ADULT PATIENTS (S7/1,2,3,4,6,8,9 AND S10>99) OR PSYCHIATRIST (S7/13) OR DIETITIAN (S6/2 AND S6B/1)

S11 In the past month, approximately how many total adults (age 18 and older) with obesity did you personally see as defined below?

Please consider all persons that had obesity, whether or not their weight was discussed during their visit.

Please use the following definition for the remainder of this survey:<br><br>A **person with obesity** is:<br>A person with a Body Mass Index (BMI) 30 or greater with or without comorbidities.

Your best estimate is fine.

[RANGE: 0-S10]

Persons with obesity in past month [_|_I_I_]

[IF QUALIFIED PHYSICIAN SPECIALTY BUT NOT PSYCHIATRIST AND SEEN AT LEAST 10 PERSONS WITH OBESITY (S7/1,2,3,4,6,8,9 AND S11/>9) OR IS A PSYCHIATRIST OR DIETITIAN AND SEEN AT LEAST 5 PERSONS WITH OBESITY (S7/13 OR (S6/2 AND S6B/1) AND S11>4) ASK S12. ELSE TERMINATE]

PHYSICIAN IN QUALIFIED SPECIALTY WHO IS NOT PSYCHIATRIST AND SEEN AT LEAST 10 PERSONS WITH OBESITY (S7/1,2,3,4,6,8,9 AND S11>9) OR PSYCHIATRIST AND SEEN AT LEAST 5 PERSONS WITH OBESITY (S7/13 AND S11/4) OR DIETITIAN AND SEEN AT LEAST 5 PERSONS WITH OBESITY (S6/2 AND S6B/1 AND S11>4)

S12 Considering all of your adult patients, what percentage do you see primarily for obesity?

|_|_|_|% [RANGE 0-100]

ALL RESPONDENTS

S15 Which of the following best describes your primary practice setting? If you practice in more than one location, please select the option that represents where you spend the majority of your time. Please select one option only.

1. Individual medical practice
2. Group practice (privately-owned but with shared facilities)
3. Group practice (employed)
4. Hospital (public or private)
5. Other

DIETITIAN WHO IS MEMBER OF SVDE AND SPENDS 50%+ OF TIME IN DIRECT PATIENT CARE (S6B_1 AND S8B_1>49)

S18 Which of the following describes your work situation?

1. Self-employed
2. Working for an Organization of Dietitians (leader)
3. Working for an Organization of Dietitians (employed)

4. Hospital (public or private)
5. Other

ALL RESPONDENTS
S20 Do you work at a specialized obesity center or clinic? (The obesity clinic may be in any practice setting - a hospital, group practice, or individual practice.)

1. Yes
2. No

DIETITIAN AND SEEN AT LEAST 5 PERSONS WITH OBESITY (S6/2 AND S11>4)
Q955 Is the care/service you provide to patients with obesity covered by compulsory health insurance?

1. Yes [CONTINUE]
2. No [NOT QUALIFIED]

ALL RESPONDENTS
S100 HIDDEN QUOTA QUESTIONS

1. **QUALIFIED PCPS** **[N=75]**
    - PHYSICIAN (S6/1)
    - SPECIALTY IS PCP: FAMILY PRACTICE OR GENERAL PRACTICE OR GENERAL INTERNAL MEDICINE WITH FOCUS ON PRIMARY CARE (S7/1-3)
    - SPENDS AT LEAST 50% OF TIME IN PATIENT MEDICAL MANAGEMENT (S8_1>49)
    - PRACTICES IN SWITZERLAND (S5/NE99)
    - IN PRACTICE 2+ YEARS (S9/2+)
    - SEEN AT LEAST 100 PATIENTS IN PAST MONTH (S10/>99)
    - SEEN AT LEAST 10 PERSONS WITH OBESITY IN PAST MONTH (S11/>9)

2. **QUALIFIED SPECIALISTS** **[N=50]**
    - PHYSICIAN (S6/1)
    - SPECIALTY IS OBSTETRICS/GYNECOLOGY, ENDOCRINOLOGY/DIABETOLOGY, PSYCHIATRY, GENERAL INTERNAL MEDICINE *WITH FOCUS ON DIABETES AND/OR OBESITY*, (S7/4,8,9,13), OR BARIATRIC SURGERY AND MANAGES PATIENTS WITH OBESITY (S7/6 AND S7B/1)
    - SPENDS AT LEAST 50% OF TIME IN PATIENT MEDICAL MANAGEMENT (S8_1>49)
    - PRACTICES IN SWITZERLAND (S5/NE99)
    - IN PRACTICE 2+ YEARS (S9/2+)
    - GIM WITH FOCUS ON DIABETES AND/OR OBESITY, OB/GYN, ENDO/DIAB, OR BARIATRIC SURGERY (S7/4,6,8,9) AND HAS SEEN AT LEAST 100 PATIENTS IN PAST MONTH (S10/>99)
    - GIM WITH FOCUS ON DIABETES AND/OR OBESITY, OB/GYN, ENDO/DIAB, OR BARIATRIC SURGERY (S7/4,6, 8,9) AND SEEN AT LEAST 10 PERSONS WITH OBESITY IN PAST MONTH (S11/>9), OR PSYCHIATRIST AND SEEN AT LEAST 5 PERSONS WITH OBESITY IN PAST MONTH (S7/13 AND S11/>4)

3. **QUALIFIED DIETITIANS** **[N=25]**
    - IS A MEMBER OF THE SVDE ASDD (SWISS ASSOCIATION OF DIETITIANS) (S6B/1)
    - PRACTICES IN SWITZERLAND (S5/NE99)
    - IN PRACTICE AT LEAST 2 YEARS (S9/2+)
    - SEEN AT LEAST 5 PATIENTS IN PAST MONTH WHO HAVE OBESITY (BMI OF 30+) (S11/>4)
    - SPENDS AT LEAST 50% OF TIME IN PATIENT CARE (S8B_1>49)
    - MANDATORY BASIC HEALTH INSURANCE COVERS THEIR SERVICES (Q955/1)

4. **UNQUALIFIED HCPS** **[N=99999]**

ALL RESPONDENTS
S105 SOFT QUOTA FOR OBESITY SPECIALISTS

1. **OBESITY SPECIALIST** **[N=20]**
    - QUALIFIED HCP (S100/1-2)
    - 50% OR MORE PATIENTS SEEN FOR OBESITY/WEIGHT MANAGEMENT (S12/>49%) **OR**
    - WORKS IN OBESITY CENTER/CLINIC (S20/1)

QUALIFIED DIETITIANS (S100/3)
S107 SOFT QUOTA FOR DIETITIANS SETTING

1. INDEPENDENT (S18/1) [N=99]
2. PART OF AN ORGANIZATION OF DIETITIANS (S18/2,3) [N=99]
3. PART OF AN HOSPITAL (S18/4) [N=99]
4. OTHER (S18/5) [N=99]

ALL RESPONDENTS
S110 SPECIALTY SOFT QUOTAS

SAMPLING SHOULD BE DONE TO ENSURE REPRESENTATION ACROSS SPECIALTIES.

Specialty	Switzerland
Family Practice (S100/1 and 7/1)	Max n=75
General Practice (S100/1 and S7/2)	Max n=75
General Internal Medicine with focus on primary care (S100/1 and S7/3)	Max n=75
**PCPs Total**	**n=75**
OB/GYN (S100/2 AND S7/8)	Max n=8
Endocrinology/Diabetology (S100/2 AND S7/9)	Max n=20
General Internal Medicine (with focus on diabetes and/or obesity) (S100/2 AND S7/4)	Max n=12
Psychiatry (S100/2 AND S7/13)	Max n=5
Bariatric Surgeons (S100/2 AND S7/6)	Max n=5
**Specialists Total**	**n=50**
Dietitians (S100/3)	**n=25**
**TOTAL HCPs**	**n=150**

ALL RESPONDENTS
S115 OBESITY SPECIALIST SOFT QUOTAS

1. Obesity Specialist (S105/1) n=999
2. Not Obesity Specialist (S105/NE1) n=999

SECTION 100: PATIENT WEIGHT HISTORY / DEMOGRAPHICS

ALL RESPONDENTS
Q100 Thank you for your responses. You have qualified for this study. Considering your entire patient population, what proportion of your patients fall into each of the following groups? If none fit into a category, please enter "0" (zero).

Your best estimate will do. Your responses should sum to 100%.

[INSERT CONSTANT SUM INDICATOR. TOTAL MUST SUM TO 100]
[RANGE 0-100]

1. Not overweight (BMI < 25) |_|_|_| %
2. Overweight (BMI 25 to 29.9) |_|_|_| %
3. Obesity class I(BMI 30 to 34.9) |_|_|_| %
4. Obesity class II (BMI 35 to 39.9) |_|_|_| %
5. Obesity class III (BMI 40+) |_|_|_| %

SECTION 200: READINESS TO CHANGE / PREVIOUS SUCCESS

PwO: Q205
ALL RESPONDENTS
Q215 As a reminder, throughout this survey, we are defining persons with obesity as those with a Body Mass Index (BMI) of 30 or greater with or without comorbidities.

To the best of your knowledge, what percentage of your patients with obesity have made what you consider a serious attempt to lose weight (e.g., followed a program, set goals, put their mind to it, or worked with a qualified healthcare professional), whether or not it was successful?

Please provide your best estimate.
[RANGE 0-100]
Percentage of patients with obesity that have made a serious attempt to lose weight |_|_|_|%

ALL RESPONDENTS
Q217 In general, what percentage of your patients who made a serious attempt to lose weight within the past year would you define as successful?

Your best estimate will do.

[RANGE 0-100]
Percentage of patients |_|_|_|%

PwO: Q203
ALL RESPONDENTS
Q225 In your experience, which of the following most motivates people to lose weight?

Select all that apply.

[MULTIPLE RESPONSE, RANDOMIZE WITHIN GROUPS. RANDOMIZE GROUP ORDER, SELECT ALL THAT APPLY], DON'T SHOW HEADINGS]

Physical Health

1. Having general health concerns
2. Wanting to stop or not need to take medication for a weight-related health condition
3. Reaching the upper end of the weight range they are comfortable with
4. A specific, personal medical event (heart attack, stroke, etc.) or diagnosis (diabetes, liver disease, sleep apnea, etc.)

Support

5. Encouragement, support, recommendations from family or friends
6. Encouragement, support, recommendations from a healthcare provider
7. Encouragement, support, recommendations from wellness / fitness programs or a personal trainer
8. Encouragement, support from others who are trying to lose weight (losing weight with a spouse, health or fitness competition, etc.)

Appearance

9. Wanting to fit into a smaller clothing size
10. Wanting to be more fit / in better shape

Goals

11. Wanting to feel better physically, have more energy or be more active
12. Wanting to be more confident / improve their self-esteem
13. Wanting to improve job performance
14. Wanting to improve their sex life
15. Wanting to be a positive role model for family / children

Life Events

16. A major life change such as retirement, divorce, break-up, starting a family
17. An upcoming special occasion or event
18. A specific medical event (heart attack, stroke, etc.) or diagnosis (diabetes, liver disease, sleep apnea, etc.) in a family member / close friend

19. Other [ANCHOR]
20. None of the above [EXCLUSIVE, ANCHOR]

ALL RESPONDENTS

Q235A For what proportion of your patients with obesity do you actually discuss their weight?

[RANGE 1-100]

|_|_|_|% of patients with obesity with whom I discuss their weight

ALL RESPONDENTS

Q240 For quality control purposes, select "Completely agree" from the list of options below.

1. Completely disagree
2. Somewhat disagree
3. Neutral
4. Somewhat agree
5. Completely agree

SECTION 400: OBESITY AWARENESS AND PERCEPTIONS

PwO: Q405
ALL RESPONDENTS
Q405 In general, how large of an impact do you believe the following health conditions have on a person's overall health?

Use a scale where 1 means "Very little impact" and 5 means "An extreme impact."

1 - Very little impact 2 3 4 5 - An extreme impact

[RANDOMIZE]
6. Diabetes
7. Chronic obstructive pulmonary disease (COPD)
8. Cancer
9. Stroke
10. Obesity (BMI of 30 or greater)

ALL RESPONDENTS
Q405A HIDDEN QUESTION
[RECODE Q405 ATTRIBUTES 1-4 BASED ON IF THEY'RE GREATER OR LESS THAN OBESITY (Q405_5)]

OBESITY IS MORE SERIOUS (Q405_ATTRIBUTE < Q405_5)
OBESITY IS AS SERIOUS (Q405_ATTRIBUTE = Q405_5)
OBESITY IS LESS SERIOUS (Q405_ATTRIBUTE > Q405_5)

ALL RESPONDENTS
Q410 Compared to a person who is not overweight, how easy or difficult do you think each of the following is for someone who is overweight?

[RANDOMIZE]	Much harder	Somewhat harder	About the same	Somewhat easier	Much easier
Getting a job	1	2	3	4	5
Advancing/promotion in a job	1	2	3	4	5
Making friends	1	2	3	4	5
Forming romantic relationships	1	2	3	4	5

ALL RESPONDENTS
Q415 How much of an impact do you think a person being overweight has on each of the following?

[RANDOMIZE]	Very negative impact	Somewhat negative impact	No impact	Somewhat positive impact	Very positive impact
How smart people think the person is	1	2	3	4	5
How athletic people think the person is	1	2	3	4	5
How healthy people think the person is	1	2	3	4	5

Relationships with friends	1	2	3	4	5
Relationships at home/with family	1	2	3	4	5
How much ambition people think the person has	1	2	3	4	5
How much willpower people think the person has	1	2	3	4	5

SECTION 500: OBESITY ATTITUDINAL QUESTIONS

PwO: Q500

ALL RESPONDENTS

Q503 Thinking of your patients with obesity as a whole, please indicate how much you agree with each of the following…

Use a scale where 1 means "Do not agree at all" and 5 means "Completely agree".

1 - Do not agree at all 2 3 4 5 - Completely agree

[RANDOMIZE,CAROUSEL]

1. It is easy for my patients to lose weight.
2. My patients could lose weight if they really set their mind to it.
3. If my patients lost weight, it would be easy for them to keep the weight off.
4. My patients know what they need to do to lose weight.
5. I have a responsibility to actively contribute to my patients' weight loss effort.
6. My patients' weight loss is completely their responsibility.
7. For my patients to lose weight, they would need to completely change their lifestyles.
8. My patients are happy with their current weight.
9. My patients are past the point where they can lose weight on their own.
10. My patients are motivated to lose weight.
11. For quality control purposes, please select 1.
12. Obesity is less important than many of the other diseases I treat.
13. I do not feel comfortable bringing up a patient's weight unless they mention it first.
14. There is nothing I can do to help patients manage their weight.
15. Treating patients with obesity is a productive use of my time.
16. I feel motivated to help patients with obesity lose weight.
17. I support/empower my patients with obesity to make healthy changes.
18. My patients know how to keep the weight off

PwO: Q507

ALL RESPONDENTS

Q507 How much do you agree that each of the following is a barrier to your patients losing weight?

Please use a scale where 1 means "Do not agree at all" and 5 means "Completely agree".

1 - Do not agree at all 2 3 4 5 - Completely agree

[RANDOMIZE, CAROUSEL]

1. Their preference for unhealthy food

2. Lack of exercise
3. Their genes
4. The nature of their job / employment
5. A lack of time to cook healthy meals
6. Their other health conditions
7. Their friends and family
8. Me, their healthcare provider
9. Their finances
10. Their lack of motivation
11. Their lack of ability to control their hunger
12. The cost of healthy food
13. Limited access to healthy food
14. Their mental health/emotional state
15. Fear of failure
16. Limited coverage for health care costs
17. Limited mobility due to physical health problems
18. The possibility of regaining the weight
19. Their unhealthy eating habits (large portions sizes, excessive snacking)
20. Their lack of understanding of what obesity is
21. The cost of obesity medications, programs and services
22. Their metabolism
23. Their age
24. Their primary care physician [DISPLAY FOR DIETITIANS ONLY (S100/3)]

Answer list is same as Q210A in PwO (methods discussed) and repeats in HCP at 515

ALL QUALIFIED

Q128 Still thinking of your patients with obesity, for what percentage do you **recommend** each of the following methods for weight management when discussing their weight (whether or not the patient followed your recommendation)?

Your responses may sum to more than 100% to account for instances where you may make more than one treatment recommendation.

[RANGE 0-100, RANDOMIZE ROWS WITHIN GROUP, RANDOMIZE GROUP ORDER EXCEPT 16 AND 99 ITEMS, DON'T SHOW HEADINGS] [NUMERIC BOX NEXT TO EACH ROW ITEM]
[MAKE NONE OF THE ABOVE EXCLUSIVE] [ANCHOR]

Diet / Healthy Eating

1. General improvement in eating habits / reducing calories
2. Specific diet or diet program (for example InShape Biomed, Weight Watchers, Metabolic Balance, Pronokal)
3. Elimination diets (avoiding fats, sugary beverages, carbohydrates, etc.)

Exercise

4. Generally be more active / increase physical activity
5. A formal exercise program / Gym membership / Personal trainer

Tracking

6. Meal / nutrient tracking (on paper or an app)

7. Exercise tracking (on paper or app such as smartphone apps, wearable fitness tracker, etc.)

Medical Treatment / Medication

8. Over-the-counter (non-prescription) weight loss medication (vitamins, supplements, etc.)
9. Prescription weight loss medication
10. Visiting a dietitian (non-physician) [DO NOT DISPLAY FOR DIETITIAN S100/3]
11. Visiting an obesity specialist
12. Behavior therapy or psychotherapy such as counseling or behavior modification
13. Weight loss surgery / bariatric surgery

Quality of life management

14. Stress management
15. Sleep quality management
16. Other [ANCHOR]
17. None of the above [EXCLUSIVE][ANCHOR]

PwO: Q210D

ALL QUALIFIED

Q515 Which of the following do you believe are **most effective for long-term weight management**?

Please select all that apply.

[MULTI SELECT]
[INSERT ENTIRE LIST FROM Q128. SHOW IN SAME ORDER AS Q128]

Diet / Healthy Eating

1. General improvement in eating habits / reducing calories
2. Specific diet or diet program ((InShape Biomed, Weight Watchers, Metabolic Balance, Pronokal)
3. Elimination diets (avoiding fats, sugary beverages, carbohydrates, etc.)

Exercise

4. Generally be more active / increase physical activity
5. A formal exercise program / Gym membership / Personal trainer

Tracking

6. Meal / nutrient tracking (on paper or an app)
7. Exercise tracking (on paper or app such as smartphone apps, wearable fitness tracker, etc.)

Medical Treatment / Medication

8. Over-the-counter (non-prescription) weight loss medication (vitamins, supplements, etc.)
9. Prescription weight loss medication
10. Visiting a dietitian (non-physician) [DO NOT DISPLAY FOR DIETITIAN S100/3]
11. Visiting an obesity specialist
12. Behavior therapy or psychotherapy such as counseling or behavior modification
13. Weight loss surgery / bariatric surgery

Quality of life management

14. Stress management
15. Sleep quality management

Other [ANCHOR]

100. I do not think any of these methods are effective for weight loss [EXCLUSIVE][ANCHOR]

PwO: Q520

ALL RESPONDENTS

Q521 Please indicate how much you agree with the following regarding prescription medications for weight loss.

Use a scale where 1 means "Do not agree at all" and 5 means "Completely agree".

[RANDOMIZE, CAROUSEL]

1 - Do not agree at all 2 3 4 5 - Completely agree 99 Don't know/ Not sure

1. Prescription weight loss medications are more effective for my patients than other treatment options for weight loss.
2. I am likely to prescribe new prescription weight loss medications. [DO NOT DISPLAY FOR DIETITIAN S100/3]
3. I am more likely to recommend my patients take a prescription medication than have a surgery (bariatric) to lose weight.
4. I am concerned about the side effects associated with prescription weight loss medications.
5. My patients would like me to offer prescription weight loss medication to help them with their weight loss efforts. [DO NOT DISPLAY FOR DIETITIAN S100/3]
6. There are good options available today for prescription weight loss medications.
7. Cost is a major barrier for my patients to consider prescription weight loss medications.
8. Patients would rather lose weight on their own than depend on medication.
9. I don't know enough about prescription weight loss medications to feel comfortable prescribing them to my patients with obesity. [DO NOT DISPLAY FOR DIETITIAN S100/3]

PwO: Q525

ALL RESPONDENTS

Q525 Please indicate how much you agree with the following regarding weight loss surgery.

Weight loss surgery is also known as bariatric surgery.

Use a scale where 1 means "Do not agree at all" and 5 means "Completely agree".

[RANDOMIZE, CAROUSEL]

1 - Do not agree at all 2 3 4 5 - Completely agree 99 Don't know/ Not sure

1. Weight loss surgery is more effective than other treatment options for weight loss.
2. Most of my patients would rather have weight loss surgery than change their lifestyle to lose weight.
3. My patients often have concerns about the safety of weight loss surgery.
4. Cost is a major barrier for my patients considering weight loss surgery.
5. The wait time for surgery is too long.
6. I would rather motivate my patients to lose weight with diet and exercise than recommend weight loss surgery.
7. Having weight loss surgery is the "easy" way out for the patient.
8. Weight loss after surgery is often not maintainable by patients.

SECTION 600: SUPPORT STRUCTURE

PwO: Q826

ALL RESPONDENTS

Q602 What are the **top 5 types of support that would be most helpful** for your patients to be successful with managing their weight?

Select your top 5.

[5 SELECTIONS] [RANDOMIZE]

1. Resources for family and friends to help understand how to be supportive
2. Specific meal plans to follow for weight management
3. Online support groups for those trying to lose weight
4. Local in-person support groups for those trying to lose weight
5. Motivational programs to help people stay on track with weight loss plan
6. More programs offered at workplaces to help people lose weight
7. Encouragement from friends/family to increase desire to keep going
8. Financial support for healthy choices (gym membership, healthy foods)
9. Diary for tracking weight over time (paper based or electronic)
10. Diary for tracking food intake (paper based or electronic)
11. Diary for tracking physical activity (paper based or electronic)
12. App with weight loss tracking and ideas for healthy eating and physical activity
13. Programs for physical activity
14. Prescription drugs for weight loss
15. Over-the-counter drugs for weight loss
16. Personal trainer / weight loss counselor
17. Weekly follow-up with a healthcare provider
18. Meetings with dietitian (non-physician)

19. A work culture that encourages a healthy lifestyle
20. Access to mental health support
21. Access to stress management support
22. Access to a physician who specializes in obesity
23. Bariatric surgery
24. Other [ANCHOR]

PwO: Q640
ALL RESPONDENTS
Q650 Please indicate how much you agree with the following statements regarding obesity and weight management:

Use a scale where 1 means "Do not agree at all" and 5 means "Completely agree".

1 - Do not agree at all 2 3 4 5 - Completely agree 99 - Does not apply

[RANDOMIZE]

1. Maintaining a healthy weight is a priority for our country's healthcare system.
2. Cost of obesity therapy / treatment is a barrier for patients to lose weight.
3. I feel the healthcare system (doctor's offices, hospitals, etc.) is a good resource for those looking to lose weight.
4. Employers play an important role in managing patients' weight.
5. Obesity is a chronic disease.
6. A loss of 5-10% body weight would be extremely beneficial to the overall health of a patient with obesity.
7. The treatment of obesity should be a team effort between different healthcare professionals.
8. For quality control purposes, please select 3.

SECTION 700: INTERACTION WITH PATIENTS

PwO: Q701/Q702
ALL RESPONDENTS
Q702 When discussing obesity with a patient, what percentage of the time do you bring it up, and what percentage of the time does the patient bring it up?

[INSERT CONSTANT SUM INDICATOR. TOTAL MUST SUM TO 100]

[RANGE 0-100]

1. I start the conversation |_|_|_|%
2. The patient starts the conversation |_|_|_|%

ALL RESPONDENTS
Q704 What are the top-5 most important criteria that you consider in order to determine whether or not you will initiate a discussion with a patient about obesity?

Please select only 5 items.

[MULTISELECT, RANDOMIZE]

1. Patient's BMI
Patient's weight
Patient's vital signs (i.e., blood pressure, heart rate)
Patient is at risk of developing new/additional obesity-related comorbidities
Patient has obesity-related comorbidities
Patient will need to take medication for obesity-related comorbidities if they do not lose weight
Patient's weight has increased since previous appointment(s)
My relationship with the patient
How receptive I think the patient will be to discussing their weight
Financial considerations for my practice (able to bill for obesity diagnosis and treatment)
How much time I have for the visit
Patient's mental state
Patient's waist circumference
Other [ANCHOR]

ALL RESPONDENTS
Q705 How comfortable are you in having discussions with your patients about their weight?

1. Not at all comfortable
2. A little comfortable
3. Somewhat comfortable
4. Very comfortable
5. Extremely comfortable

PwO: Q770
ALL RESPONDENTS
Q708 What are the **top 5** reasons for which you **might not** discuss obesity with a patient?

Please select up to 5 items only

[MULTISELECT 5 ANSWERS, RANDOMIZE]

3. The appointment is not long enough / I'm rushed
4. There are more important health issues/concerns to discuss
5. I do not feel comfortable bringing it up
6. I do not trust and/or do not have a close relationship with my patient
7. I do not see weight as a significant medical issue
8. Patient is in good health and does not have weight-related comorbidities
9. I believe it is the patient's responsibility to manage their weight
10. Patient is not interested in losing weight
11. Patient does not feel motivated to lose weight
12. Patient does not believe he/she is able to lose weight
13. Even if the patient were to lose weight, he/she would just gain it back
14. Patient already knows what he/she needs to do to manage their weight
15. There is nothing I can do to help patients managing their weight
16. I am not interested enough in/concerned enough about patients' weight
17. I have had previous bad experience discussing weight with a patient
18. Patient does not have financial means to support a weight loss effort
19. I do not have training to provide weight management services

20. My office is not set up to treat patients with obesity
21. I do not get financial compensation for treating obesity
22. Other [ANCHOR]

PwO: Q720

ALL RESPONDENTS

Q720 What types of weight management goals do you set with your patients with obesity?

Please select all that apply.

[MULTISELECT] [RANDOMIZE]

1. Maintain current weight without gaining more
2. To lose (any amount of) weight
3. To lose a pre-specified % of body weight
4. To lose a pre-specified number of kilograms
5. To decrease the number of medications they must take
6. To improve their existing health condition(s)
7. To reduce the risks associated with weight / prevent a health condition
8. To have more energy
9. To improve their appearance
10. Short-term (within the next six months) individual weight loss goals
11. Long-term (more than six months from now) individual weight loss goals
12. To improve their lifestyle
13. To reduce their stress and improve overall health and well-being
14. To improve their physical and mental health and well-being
15. Other [ANCHOR]
16. I do not set weight management goals with my patients. [EXCLUSIVE, ANCHOR]

ALL RESPONDENTS

Q734 Is an obesity diagnosis typically recorded in the patient's medical record/journal?

26. Never
27. Rarely
28. Sometimes
29. Most of the time
30. Always

ALL RESPONDENTS

Q734B In what proportion of your patients with obesity do you inform them that they have a diagnosis of obesity?

[RANGE 1-100]

Inform the patient about the diagnosis of obesity |_|_|_|%

[ ] I never inform the patient about the diagnosis of obesity [EXCLUSIVE]

PwO: Q759

ALL RESPONDENTS

Q740A For what proportion of your patients with obesity do you schedule a **follow-up appointment** to discuss their weight?

Please enter '0' if you don't schedule follow up appointments to discuss their weight.

[RANGE 0-100]
|_|_|_|% of patients with obesity for whom I schedule a **follow-up appointment** to discuss their weight

PwO: Q760
SCHEDULES FOLLOW UPS WITH PATIENTS (Q740A/>0)
Q740 How often do patients with obesity **keep their follow-up appointment**?

1. Never
2. Rarely
3. Sometimes
4. Most of the time
5. Always

DIETITIANS (S100/3)
Q790 What proportion of your patients with obesity are referred to you by each of the following?

Your best estimate is fine. Your responses must sum to 100%.
[SHOW CONSTANT SUM INDICATOR, MUST SUM TO 100%] [RANGE 0-100]

1. Primary care physician |_|_|_|%
2. Obesity specialist |_|_|_|%
3. Specialist (Endocrinologist, OB/GYN, Gastroenterologist, Cardiologist, etc.) |_|_|_|%
4. The patient themself |_|_|_|%
5. Someone else |_|_|_|%

DIETITIANS / (S100/3)
Q800 What is the typical amount of time a patient with obesity is under your care?

1. Less than 2 weeks
2. 2 weeks to less than a month
3. One to two months
4. 3-5 months
5. 6-9 months
6. 10-11 months
7. 1 year
8. 2 years or more

DIETITIANS (S100/3)
Q807 How much impact do each of these factors have on how frequently you meet with a patient with obesity?

Please use a scale where 1 means "No impact at all" and 5 means "A great deal of impact".

1 – No impact at all 2 3 4 5 – A great deal of impact

[RANDOMIZE]
1. Insurance coverage (how many visits will be covered or limitation on frequency)
2. Patient preference
3. Recommendation from the referring HCP
4. The patient's level of obesity (overweight, Class 1, Class II, Class III)
5. Your own availability / workload
6. Other [ANCHOR]

ALL RESPONDENTS
Q810 Please indicate how much you agree with each of the following statements.

Please use a scale where 1 means "Do not agree at all" and 5 means "Completely agree".

1 - Do not agree at all 2 3 4 5 - Completely agree

[RANDOMIZE]
1. More patients with obesity would benefit from counseling of a dietitian
2. Physicians should refer patients with obesity to dietitians more often than they currently do.
3. I already collaborate with a dietitian for my patients.
4. Physicians should refer patients with obesity to a dietitian early/sooner in the treatment process once obesity is identified.
5. Physicians should refer patients to a dietitian *before* they are diagnosed with obesity -- when the patient is overweight.
6. There is adequate collaboration between physicians and dietitians in the care of patients with obesity.
7. Referral to a dietitian t should be one of the first methods of treating obesity (before prescription medication or bariatric surgery).
8. Dietitians are seen as valuable and respected partners by other healthcare providers in the treatment of people with obesity.
9. The wait time from referral to the first visit with a dietitian for a patient with obesity is acceptable.
10. Dietitians should have more say in treatment decisions for patients with obesity.

ALL RESPONDENTS
Q750 [FOR PHYSICIANS (S100/1,2): For which of the following reasons might you refer a patient with obesity for specialized obesity management?] [FOR DIETITICANS (S100/3): For which of the following reasons might you recommend specialized obesity management for a patient with obesity?]

Please select all that apply.

[MULTISELECT, RANDOMIZE]

23. The patient asks to see a specialist
24. The patient had been unable to achieve their weight loss goals under previous treatment plan
25. The patient's weight is related to another health condition they have
26. I do not feel confident in providing the patient the advice they need to be successful
27. They have been unsuccessful in achieving their goals under my advice
28. Coverage of weight-management related care (e.g. psychologist, dietitian, non-physician nutritionist, etc.)
29. Their financial status
30. I do not feel comfortable discussing the patient's weight [DO NOT DISPLAY FOR DIETITIANS S100/3]
31. Initiation of pharmacotherapy

32. Indication for bariatric surgery
33. Availability of a specialized weight loss program
34. Other [ANCHOR]
35. I do not refer patients to obesity specialists [EXCLUSIVE, ANCHOR]

ALL QUALIFIED
Q759D Which of the following clinical treatment guidelines do you follow for treating patients with obesity?

Please select all that apply.

1. Local (for example institution's own guidelines)
2. National (Adipositas-Consensus of the ASEMO/SGED)
3. International (e.g., European Association for the Study of Obesity (EASO), American Heart Association / American College of Cardiology / Obesity Society Clinical Practice Guideline)
4. I do not follow clinical treatment guidelines when treating patients with obesity

ALL RESPONDENTS
Q758 How effective do you think current clinical guidelines for treating obesity are?

[DISPLAY AS GRID]

	Local	National	International
1. Not at all effective			
2. A little effective			
3. Somewhat effective			
4. Very effective			
5. Extremely effective			
6. Not applicable			

PwO : q825
ALL RESPONDENTS
Q760 Please select the top 3 most helpful types of information you can provide patients to support their weight loss efforts.

Please select only three items.

Information on…

[SELECT 3 ONLY]
1. Healthy ways to lose weight
2. The health benefits of weight loss
3. Medical treatment options for obesity
4. Managing weight with exercise
5. How to maintain weight loss
6. How healthcare providers can help with obesity
7. Stress management techniques
8. Realistic weight loss goals
9. Exercises which are safe to do for people with mobility limitations
10. Healthy vs. non-healthy eating

SECTION 900: DEMOGRAPHICS

ALL RESPONDENTS
Q900 Thank you again for your time so far. As we noted at the beginning of this survey, your personal information will never be shared with other organizations for any purpose. Your honest answers are very much appreciated. Your responses to this survey will help the sponsor, Novo Nordisk, understand the healthcare experiences of patients and healthcare providers.

To finish, we would like to gather some additional information used for categorization purposes.

Please select continue.

PwO: Q900
ALL RESPONDENTS
Q906 What percentage <u>of your patients with obesity</u> are diagnosed with each of the following conditions?

Your best estimate will do.

|_|_|_|% [RANGE 0-100]
[RANDOMIZE]

1. Cardiovascular Diseases (e.g., coronary artery disease, coronary heart diseases, congestive heart failure, pulmonary embolism, stroke)
2. Depression/Anxiety
3. High cholesterol (Dyslipidemia / triglycerides)
4. High blood pressure (Hypertension)
5. Infertility
6. Liver disease (e.g., non-alcoholic fatty liver disease)
7. Obstructive Sleep Apnea
8. Osteoarthritis
9. Metabolic syndrome
10. Stomach or intestinal problems
11. Pre-diabetes
12. Diabetes (Type II)
13. Cancer
14. Polycystic Ovary Syndrome (PCOS) (the % of your *female* patients with obesity)
15. Eating disorder (e.g., binge eating disorder, night eating syndrome)

ALL QUALIFIED PHYSICIANS (S100/1,2)
Q922 Do you consider yourself an expert in obesity?

1. Yes
2. No

ALL RESPONDENTS
Q925 Have you received advanced training specifically in obesity/weight management [DISPLAY FOR PHYSICIANS (S6/1): beyond medical school]?

1. Yes
2. No

ALL RESPONDENTS
Q930 Do you provide care for obesity/weight management as a primary treatment objective?

1. Yes
2. No

ALL RESPONDENTS
Q932 For how many years have you been providing obesity care/management to patients?

Your best estimate will do. If less than one year, please enter "0" (zero).

[RANGE: 0-S9]
Years providing obesity care/management to patients |_|_|

ALL RESPONDENTS
Q950 Are you part of an interdisciplinary obesity treatment team?

An interdisciplinary team including for example a primary care physician, obesity specialist, surgeon, dietician, psychologist, movement therapist, and/or nurse.

1. Yes
2. No

PwO: Q929
ALL RESPONDENTS
Q920 Which one of the following best describes the community in which your primary place of work is located?

4. City (urban center of influence)
5. Urban area close to a city
6. Rural area / village
